# On the dependent recognition of some long zinc finger proteins

**DOI:** 10.1093/nar/gkad207

**Published:** 2023-03-23

**Authors:** Zheng Zuo, Timothy Billings, Michael Walker, Petko M Petkov, Polly M Fordyce, Gary D Stormo

**Affiliations:** Department of Genetics, Stanford University, CA, USA; Department of Genetics, Washington University in St. Louis, MO, USA; The Jackson Laboratory, ME, USA; The Jackson Laboratory, ME, USA; The Jackson Laboratory, ME, USA; Department of Genetics, Stanford University, CA, USA; Chan Zuckerberg Biohub, San Francisco, CA, USA; Department of Bioengineering, Stanford University, CA, USA; Stanford ChEM-H Institute, Stanford University, CA, USA; Department of Genetics, Washington University in St. Louis, MO, USA

## Abstract

The human genome contains about 800 C2H2 zinc finger proteins (ZFPs), and most of them are composed of long arrays of zinc fingers. Standard ZFP recognition model asserts longer finger arrays should recognize longer DNA-binding sites. However, recent experimental efforts to identify *in vivo* ZFP binding sites contradict this assumption, with many exhibiting short motifs. Here we use ZFY, CTCF, ZIM3, and ZNF343 as examples to address three closely related questions: What are the reasons that impede current motif discovery methods? What are the functions of those seemingly unused fingers and how can we improve the motif discovery algorithms based on long ZFPs’ biophysical properties? Using ZFY, we employed a variety of methods and find evidence for ‘dependent recognition’ where downstream fingers can recognize some previously undiscovered motifs only in the presence of an intact core site. For CTCF, high-throughput measurements revealed its upstream specificity profile depends on the strength of its core. Moreover, the binding strength of the upstream site modulates CTCF’s sensitivity to different epigenetic modifications within the core, providing new insight into how the previously identified intellectual disability-causing and cancer-related mutant R567W disrupts upstream recognition and deregulates the epigenetic control by CTCF. Our results establish that, because of irregular motif structures, variable spacing and dependent recognition between sub-motifs, the specificities of long ZFPs are significantly underestimated, so we developed an algorithm, ModeMap, to infer the motifs and recognition models of ZIM3 and ZNF343, which facilitates high-confidence identification of specific binding sites, including repeats-derived elements. With revised concept, technique, and algorithm, we can discover the overlooked specificities and functions of those ‘extra’ fingers, and therefore decipher their broader roles in human biology and diseases.

## INTRODUCTION

**Figure 1.**
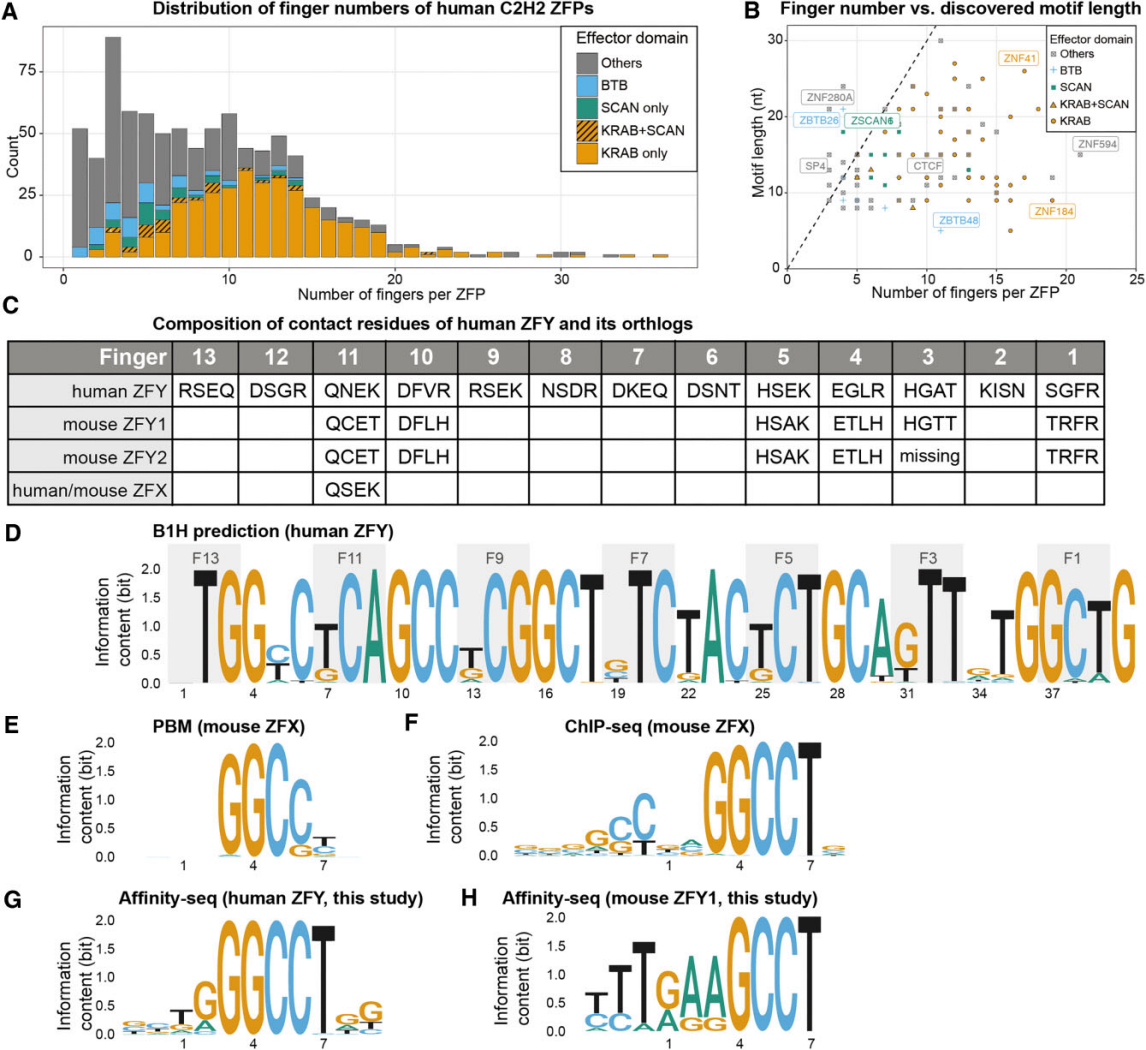
The statistics of human ZFPs and currently identified short motifs of ZFY and its orthologs. (**A**) Distribution of C2H2-type zinc finger proteins (ZFPs) in human genome. (**B**) Currently identified motif length vs. the number of fingers within each ZFP. (**C**) Contact residues of Zinc Finger Y (ZFY) and its close orthlogs, mZFY1, mZFY2 and ZFX. (**D**) Predicted motif by B1H method. (**E**) Motif of mouse ZFX identified by protein binding microarray (PBM); (**F**) Motif of mouse ZFX identified by ChIP-seq. (**G**,**H**) Motifs of human ZFY and mouse ZFY1 identified by Affinity-seq in this work respectively.

The zinc finger domain was first described in the TFIIIA protein of Xenopus, which contains an array of zinc fingers for the recognition of RNA Polymerase III promoters (1,2). Since then, it has been discovered that ZFPs exist in all eukaryotic species and have expanded enormously in the vertebrate lineage (3–5) where they are the most abundant class of transcription factors (TFs) (6,7). While ZFPs can have other roles, such as binding to RNA and in protein–protein interactions, it is generally thought that C2H2 family ZFPs function as DNA-binding TFs. Most C2H2 family ZFPs are composed of tandem arrays of fingers, and the number of fingers for each ZFP has greatly expanded in vertebrates, with some human ZFPs containing more than 30 fingers (Figure 1A). Although they have been studied for decades, there are some unresolved problems.

First, it is unclear why many long ZFPs seem to only bind short motifs. Crystal structures of ZFPs bound with DNA show that most of the fingers interact with 3–4 base pairs (bp) and form tandem triplets (8–10). Therefore, for a ZFP with N fingers, we expect it to bind a sequence 3N bp long and have a motif of that length. Consistent with this, the mainstream motif prediction method (11) predicts the full-length motif by concatenating the motifs for individual fingers derived from bacterial-one-hybrid (B1H) assays (11,12). However, motifs identified for many ZFPs by ChIP-seq and HT-SELEX (13–15) are significantly shorter than these predictions (Figure 1B). Zinc finger Y (ZFY), for example, is the only zinc finger gene located on the human Y chromosome with 13 tandem fingers (Figure 1C), it has two close homologs in mice (mZFY1 and mZFY2), which are required for the meiotic sex chromosome inactivation (MSCI) (16). ZFX, its counterpart on the X chromosome, also shares close sequence homology. The B1H prediction (11) suggests they should have a ∼39 bp long consensus binding sites (Figure 1D). However, Taylor-Harris *et al.* (17) performed SELEX on mZFY1 and found it preferentially binds to sequences containing GGCCT; Grants et al (18) showed that mZFY fingers 11–13 are sufficient to recognize a RGGCCT motif; PBM (19) and ChIP-seq (20) experiments of ZFX also produced the 5 bp GGCCT motif, though ChIP-seq result shows some extra upstream component (Figure 1E, F). We do not know whether they really recognize such short motifs or its true specificity profiles are underestimated for other reasons.

Second, it is unclear whether those seemingly unused fingers are functionally important. The genome insulator CTCF, for example, was previously shown to confer multi-mode recognition to upstream, core, and downstream motifs by distinct sets of fingers respectively, but the majority of identified *in vivo* binding sites don’t contain flanking sequences that match the upstream or downstream motifs (21), thus the function of those ‘extra’ fingers is unclear. Nonetheless, all eleven fingers are highly conserved across mammals and a missense mutant in one upstream finger was found to cause severe dementia in multiple cases (22,23). Either those upstream fingers function as an independent module, or alternatively, they exert their regulatory roles through allosteric effects onto adjacent core fingers. A quantitative assay to systematically dissect CTCF’s recognition property is required to confirm or falsify either case.

Third, if the current underestimation of motif length is caused by our incomplete understanding about the biophysical properties of long ZFPs, it is unclear how to revise the motif discovery algorithms and whether the revised model or algorithm can improve the prediction accuracy of *in vivo* binding sites. ZIM3 and ZNF343 are examples here to demonstrate the utility of combined use of fixed-core motif inference, auto-correlation analysis, and group-wise ChIP-exo footprinting to infer the recognition models of long ZFPs prior to biophysical experiment.

Our results show that the current simple, additive model alone is inaccurate and inadequate to characterize the mutually dependent, multi-mode recognition properties of long ZFPs, and the revised model and algorithm help reveal their hidden motifs, modes, functions and disease mechanisms.

## MATERIALS AND METHODS

### Construction and expression of recombinant proteins

The coding sequences for human ZFY (Uniprot P08048:408–768) and mouse ZFY1 (Unirprot P10925:390–782) were codon optimized for E. coli expression and synthesized as IDT gBlocks and mouse CTCF (Uniprot Q61164-1:241–583) was cloned from mouse cDNA libraries. After In-Fusion cloning into an NEB DHFR control vector with an N-terminal hisSUMO tag (Supplementary Figure S1), proteins were expressed and purified largely as in our previous work except that we used an extra heparin column purification to increase purity for anisotropy experiments. For Methyl-Spec-seq experiments of CTCF, we used the NEB PURExpress system to produce N-terminal HALO tagged CTCF constructs (Supplementary Figure S1) and noticed better success rates than previous SUMO-tagged constructs. All constructs, including truncated versions, are listed in Supplemental Table S1.

### Affinity-seq procedures

Affinity-seq was essentially done as in (24) with minor adjustments. A ZF array of the protein of interest was amplified then cloned into a universal Affinity-seq vector by recombineering. The resulting construct expressed a fused protein containing 6HisHALO–the 412–511 aa fragment of PRDM9–ZF array of interest. The fused protein was expressed in Rosetta 2 cells at 15°C for 24 h and partially purified by ion exchange chromatography on SP-sepharose. The purified protein was mixed with genomic DNA sheared to ∼200 bp on a Covaris ultrasonicator, and allowed to bind overnight. The protein-DNA complexes were then isolated on HisPur Ni-NTA Resin (Thermo Scientific) preincubated with a partially purified prep of the empty tag to reduce the background. DNA was then eluted and used to prepare genomic libraries using a TruSeq ChIP Library Prep Kit (Illumina). The libraries were sequenced on a HiSeq2500 or NextSeq platform ensuring ∼50 million reads per library. Data were analyzed using a custom pipeline as described previously (24) and we used the MEME (v4.10.1) software package with default parameters (*P*-value threshold 0.001 and 150 bp central peaks regions) for motif discovery.

### HT-SELEX procedures

For the first round of HT-SELEX, ∼200 ng dsDNA libraries containing randomized sequence CAGGCCTNNNNNNNN were used for EMSA shift with hisSUMO-hZFY titrated from low to high concentration. Each time, only the lane containing the lowest amount of protein was chosen and the bound portion of DNA (no more than 20% of total DNA) was cut and then amplified for the next round of SELEX selection enrichment. Since in the first round of HT-SELEX, the most enriched site turned out to be CAGGCCTAGGCGTTG, the DNA library was redesigned as CAGGCCTAGGCGTNNNNNNNN for further HT-SELEX by EMSA separation. Again, each time we ensured that no >20% of the total DNA was in the bound state for selection and enrichment analysis.

### Spec-seq, Methyl-Spec-seq procedures and motif analysis

The experimental procedures were essentially the same as our previous work (25), with all binding reactions set up at 1× NEBuffer 4, room temperature. For ZFY, EMSAs were performed using 9% Tris-glycine gels in the cold room run at 200V for 30mins. We noticed that for some ZFY proteins, particularly ZFY (F11-F13), when the protein concentration was too high, the shifted DNA fragments appeared easily to form protein oligomers or aggregate near the EMSA well. Consequently, we generally used low concentrations of protein (<100 nM) and selected only monomeric ZFY–DNA complexes for Spec-seq analysis. For CTCF, 12% Tris-glycine gels were used to separate the bound and unbound fractions of DNA (Supplementary Figure S2). Position energy matrices or energy logos were derived by data regression of the binding energy of either reference sites plus single variants or all measured sites using the TFCookbook (26) package and the analysis workflow is listed in Supplemental Table S3.

### Dissociation kinetics assay by fluorescence anisotropy

All binding assays were performed in 1× NEBuffer 4 at 37°C with 30nM FAM-DNA probe, and in this condition the basal value for FAM-DNA probe without protein was ∼15 mA. With a saturating concentration of ZFY protein added, the anisotropy values can go above 100mA (Supplementary Figure S7). In our case, we titrated a low volume of protein (<4% v/v), yielding initial values at equilibrium only above 40 mA, suggesting that only a small fraction of the DNA was bound (<20%) and the DNA probe was more likely bound by the protein in an assumed specific conformation. After we injected highly concentrated unlabeled competitor DNA (500 pM/ul × 2 ul) into 100 ul binding reactions, yielding a molar ratio between FAM probe and competitor DNA <1:200, we measured anisotropy values at 20 s or 40 s time intervals for up to 90 min.

To measure the intrinsic dissociation rates of ZFY–DNA complexes, we did titration experiments first with different molar ratios of unlabeled competitor DNA into the binding reactions, as in Supplementary Figure S5. For competition ratios below 1:100, the observed dissociation rate reached some plateau and did not increase further. Therefore, we assumed it was appropriate to use the 1:200 competitor ratio curve to estimate the intrinsic dissociation rate or mean lifetime of the protein–DNA complex.

After setting up the binding reaction for at least 20 min, we assumed the system had reached equilibrium state. Slightly to our surprise, however, we observed the anisotropy value slowly decrease over time even in the absence of any added competitor DNA, likely representing steady inactivation or degradation of ZFY protein at 37°C. To exclude the possibility that the measured dissociation rates are differentially biased by different protein inactivation rates, we measured this inactivation process alone for different proteins, and they all showed very similar inactivation rates, which are significantly slower than our observed dissociation rates (Supplementary Figure S6).

To quantify the dissociation rate *k*_off_ or mean lifetime τ, we fit our data using single exponential decay model with following equation:


$$\begin{equation*}{\rm FAM}\left( t \right) = {\rm range} \times {{\rm e}}^{ - {t \mathord{\left/ {\vphantom {t \tau }} \right. } \tau }} + {\rm base}\end{equation*}$$

The base value is usually in the range 15–17, and the range parameter depends on the first measured anisotropy value in each experiment, which should not affect the mean lifetime. For full-length ZFY construct interacting with the S.S.S. (long) probe, we noticed a significant discrepancy between observed data and fitted curves, so a two-phase exponential decay model was also used:


$$\begin{equation*}{\rm FAM}\left( t \right) = {A}_1 \times {{\rm e}}^{ - {t \mathord{\left/ {\vphantom {t {{\tau }_1}}} \right. } {{\tau }_1}}} + {A}_2 \times {{\rm e}}^{ - {t \mathord{\left/ {\vphantom {t {{\tau }_2}}} \right. } {{\tau }_2}}} + {\rm base}\end{equation*}$$

Each experiment was repeated at least three times to calculate the mean values and standard deviations (as in Supplemental Table S2). All experiments were performed using a TECAN Safire2 instrument set to 490nm excitation/525nm emission wavelength.

### Alignment and comparison of published CTCF structure models

ChimeraX was used to superimpose two structure models (PDB #5KKQ, #5YEL) based on alignment of the cytosine and guanosine at position 2.

## RESULTS

### Short motifs are obtained for hZFY and mZFY1 using Affinity-seq

To identify preferred sequences for ZFY, we leveraged Affinity-seq, a method for *in vitro* selection of fragmented genomic DNA followed by MEME motif analysis (27), to identify all bound sites from the entire genome sequence. Affinity-seq on human ZFY and mouse ZFY1 yielded 90084 and 50 170 peaks at *P* values <0.01, respectively, from which we found motifs very similar to those previously reported (Figure 1G,H), with no secondary motifs reported by MEME.

### High-throughput SELEX (HT-SELEX) reveals a downstream consensus site and Spec-seq confirms this irregular downstream motif by fingers 7-11 of ZFY

**Figure 2.**
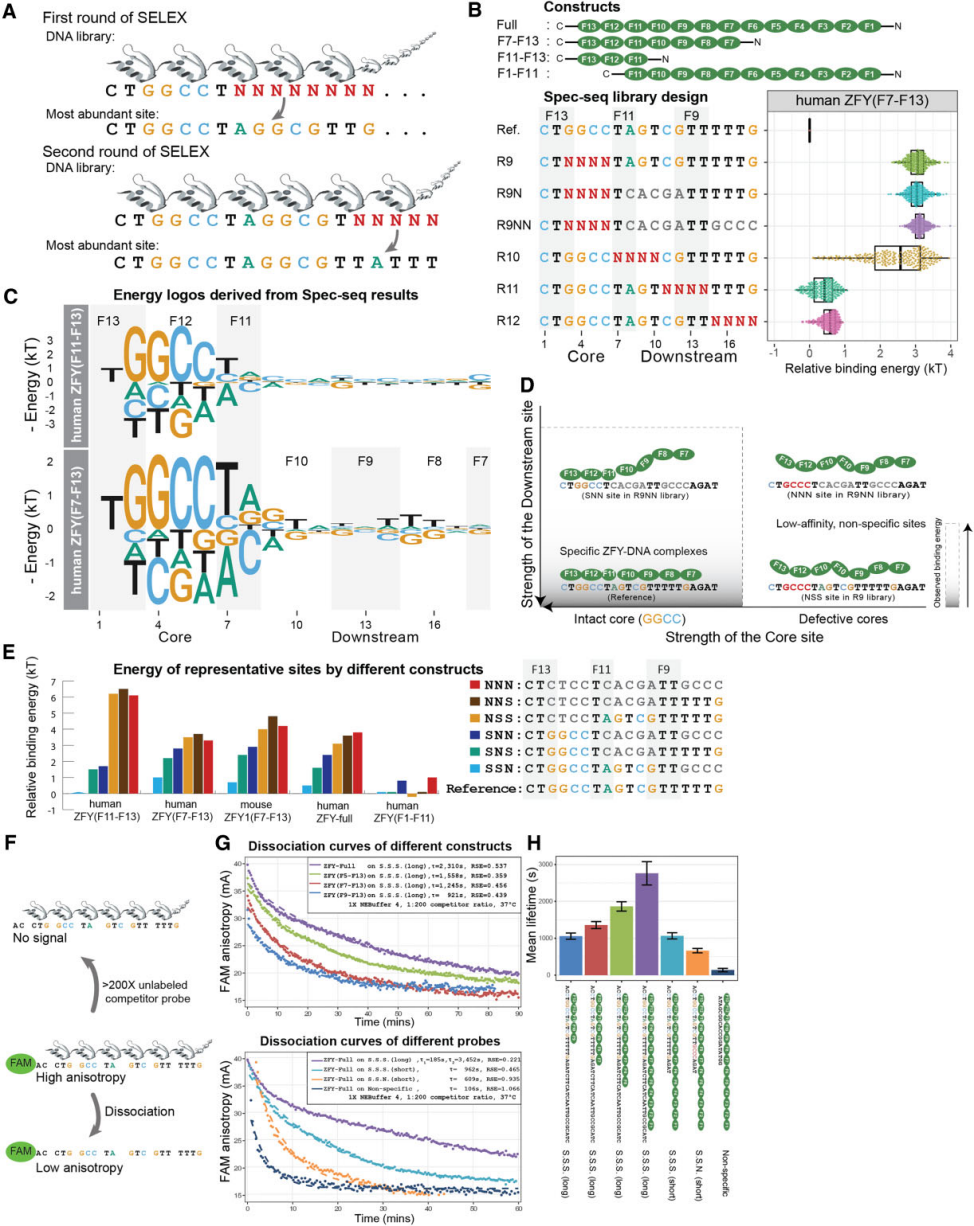
Quantitative analysis of ZFY reveals that its downstream recognition depends on the presence of perfect core. (**A**) Workflow of two rounds of HT-SELEX with previously identified binding site as the anchor position. (**B**) Tested constructs, Spec-seq library design and the variants distribution of observed binding energy under human ZFY (F7-F13) construct. (**C**) Position energy logo constructed by regression of all single variants of reference site. (**D**) Dependent recognition model for Zinc Finger Y (ZFY); Without intact core, there is no observed specificity towards downstream region. (**E**) Energy levels for representative binding sites under different constructs; S.S.N. is short name for Specific-Specific-Non-Specific, and so on. (**F**) Workflow of florescence anisotropy assay to measure the intrinsic dissociation rates of various protein-DNA complexes; >200X unlabeled competitor probe was added to the pre-equilibrated binding reaction right before the kinetics monitoring processes. (**G**) Upper panel shows dissociation curves for full-length ZFY over various DNA probes; Lower panel shows dissociation curves for full-length and various truncated ZFY constructs over S.S.S. (long) DNA probe; Single phase exponential curve was used to fit all observed data, except the ZFY-Full over S.S.S. (long) in lower panel, which shows two-phase exponential curve; Residual Standard Error (RSE) were listed on he upper right panels. (**H**) Summary of observed dissociation rates of different constructs over various probes; Each sample was measured for at least three times; Sample means and standard deviations were derived from fitted single exponential curves.

To test whether ZFY has any extended motif beyond GGCCT, we adopted High-throughput SELEX (HT-SELEX) (13), using randomized dsDNA libraries with a prefixed GGCCT in the flanking region (Figure 2A). Since the initial pool is made of degenerate oligos, we expect it to be close to random pool. After two rounds of bound DNA selection by EMSA separation and amplification, we sequenced the enriched DNA pool and found the most abundant site in the pool to be GGCCTAGGCGTTG. We then fixed that extended consensus site, extended the randomized dsDNA region, and reran the SELEX assay, obtaining a most abundant site of GGCCTAGGCGTTATTTT in the new pool (Figure 2A). In literature, most of ZFP motif predictions were arranged from C- to N-end of proteins, so we kept that convention, and the upstream (downstream) notations are defined with the same orientation as the predicted ZFP-DNA complexes.

Compared with other high-throughput techniques like HT-SELEX and Affinity-seq, Spec-seq (28) is a medium-throughput method to quantitatively characterize the energy landscape of TF–DNA interactions with energetic resolution down to 0.2*k*_B_*T*. One practical limitation of Spec-seq is that one can only assay the relative binding energy up to a few thousands of variants, so having prior knowledge about the consensus site is preferable.

Using this SELEX-enriched site as a starting consensus sequence, we constructed tandem, non-overlapping dsDNA libraries for pilot Spec-seq runs and found that GGCCTAGTCGTTTTTG had slightly higher affinity than the SELEX-enriched site, which is not significantly more abundant than similar sequences; therefore, we chose this sequence as the reference site for following runs. We designed four randomized dsDNA libraries (Rand 9, 10, 11, 12) to tile across the entire reference site (Figure 2B, upper panel) and used Spec-seq to quantify relative binding energies for full-length mouse ZFY1, full-length human ZFY and truncated versions of human ZFY containing different subset of ZFs (F11–F13, F9–F13, F7–F13, F5–F13, F1–F11). Consistent with previous work, we observed ∼0.2*kT* measurement variation for individual sequences between replicate runs (Supplementary Figure S3), defining a practical resolution limit for significance.

Then we built position energy matrices (PEMs) and corresponding logos based on regression of the energy values of the chosen reference site and all its single variants (Figure 2C). Compared to the hZFY (F11–F13), the motif for hZFY (F7–F13) revealed a downstream preference for a GT—TTT, indicating that fingers F7–F10 contribute to the recognition at positions 9–18. Since B1H method predicts that F12–F13 are responsible for the recognition at positions 1–6 (Figure 1D), it is very likely that finger 11 only recognizes a 2nt long TA motif in positions 7–8 rather than the predicted 3nt TCA. These results show that there exists some specific configuration of ZFY–DNA complex with at least seven fingers engaged in the recognition, and both the core and downstream regions contribute to its total binding energy (Figure 2D).

### Recognition of downstream sites by ZFY depends on an intact core

Next, we compared the binding energy of variants in the Rand9 library (R9) to two extra libraries (R9N, R9NN) containing the same randomized sequences within the core but some mismatches downstream (Figure 2B, lower panel). All variants in the R9, R9N, R9NN libraries excluding the reference site fall within the non-specific, plateau range (3 kT above reference), regardless of downstream sequences. Most likely, single mismatch within the core region destabilizes the assumed specific complex so much that the even the strongest downstream site couldn’t compensate the affinity loss to form a high-affinity complex (Figure 2D), thus it is of no practical value to use the original motif derived from single variants data to predict the energy of those sites with defective cores anymore (Supplementary Figure S4).

To validate this model, we quantified binding of various full-length and truncated constructs (hZFY (F1–F11), hZFY (F11–F13), hZFY (F7–F13), mZFY1 (F7–F13) and full-length hZFY) to a few representative sequences within tested libraries (Figure 2E). For example, the site CTGGCTAGTCGTTGCCC contains an intact core, a specific downstream site at position 7–12, and a non-specific downstream site at 13–18, which we designate as specific–specific–non-specific, or SSN for short. Under this naming scheme, SSS is the reference site and set as the baseline for comparison between different constructs. While hZFY (F11–F13), hZFY (F7–F13), mZFY1 (F7–F13) and hZFY-full clearly show similar relative patterns of recognition to sites containing an intact core (i.e. the SSS reference sequence is bound more strongly than SSN, SNS, and SNN sites due to preferential recognition by downstream fingers), there is no consistent difference between measured energies for NXX sites (i.e. NSS, NNS and NNN). Additionally, for hZFY (F1–F11) which lacks the fingers for cores recognition, the observed energetic differences between SSS, SNS, SSN, NSS, NNS are below the usual reproducibility limits of Spec-seq experiment (0.25 kT, Supplementary Figure S3, Figure 2E). Together, these results demonstrate that specific recognition of downstream fingers depends on the presence of intact core by fingers F11–F13 to form high-affinity complex (Figure 2D).

### Downstream fingers contribute to the stability of ZFY–DNA complexes

Besides binding energy measurement, we directly measured the intrinsic dissociation rates ${k}_{off}$ for various ZFY constructs interacting with different DNA sequences by fluorescence anisotropy. After binding reactions reached equilibrium, we added a 200-fold excess of unlabeled competitor DNA and monitored anisotropy values of the FAM-DNA probes over time to visualize dissociation processes (Figure 2F, G). Changes in mean lifetime for full-length hZFY interacting with different DNA sequences were consistent with Spec-seq measurements: SSS and SSN had a 0.7*kT* energy or 2-fold affinity difference as measured by Spec-seq, and their mean lifetimes also differed by 2-fold (1176s and 656s, respectively), confirming that measured energy differences for ZFY–DNA binding are primarily driven by differences in their dissociation rates. However, while measured dissociation curves for truncated ZFYs interacting with SSS (long) probe and full-length ZFY interacting with SSN (short) or nonspecific probes were well-fit by a single exponential curve, full-length ZFY dissociation from SSS (long) probe showed significant deviation from single exponential behavior (Figure 2G upper panel, residual standard error (RSE) 0.537). We resorted to two-phase exponential curve fitting, which yielded better results (Figure 2G, lower panel, RSE 0.221). These observations suggest that a simple two-state DNA binding model is inadequate to address the complex, multi-mode recognition of ZFP.

### Observed upstream specificity of CTCF depends on the strength of the core

**Figure 3.**
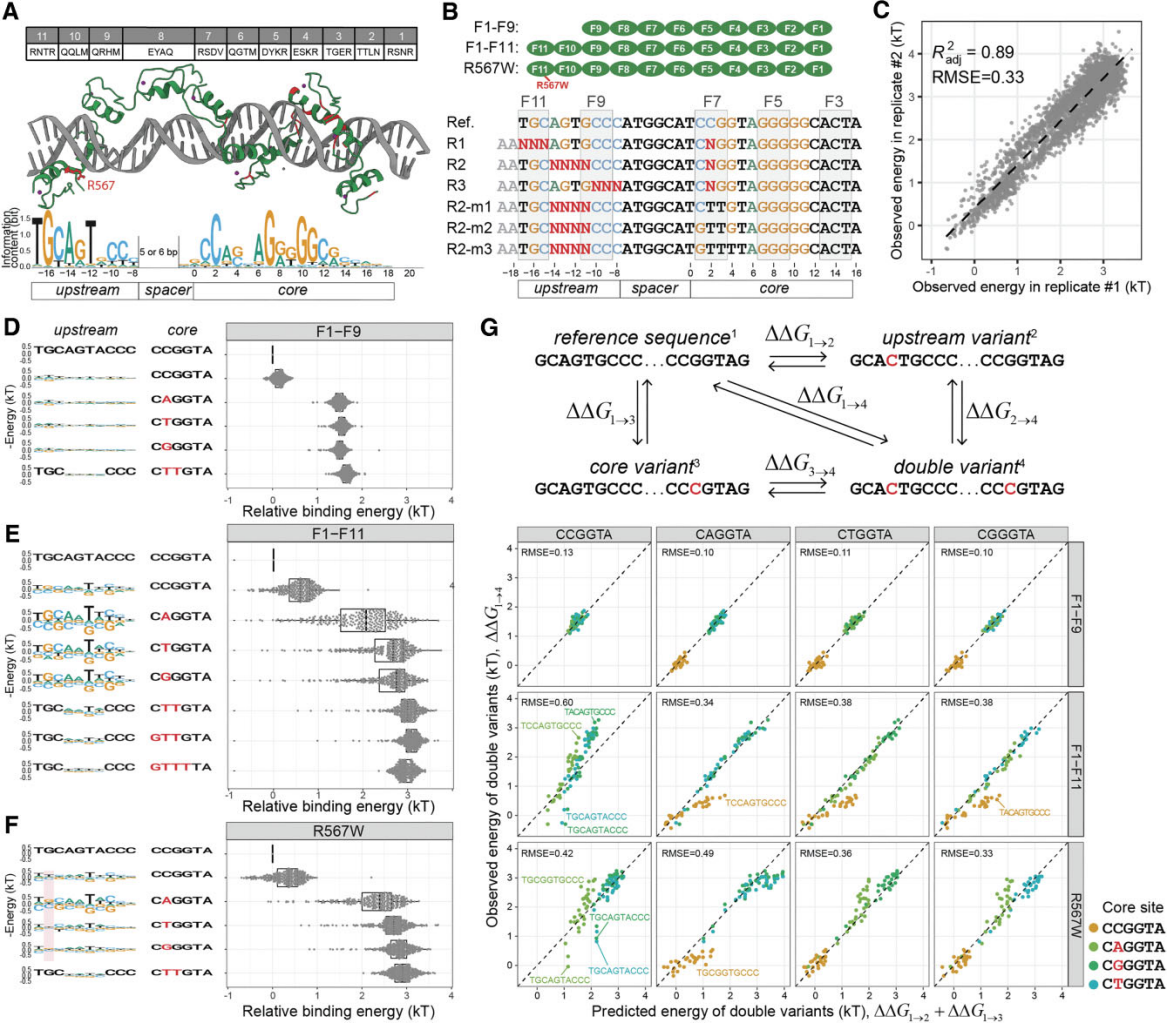
The upstream specificity of CTCF depends on the strength of core sites and violates additivity assumption. (**A**) Contact residues composition for human/mouse CTCF and current structural model about CTCF’s recognition to its binding site. Currently identified missense disease mutants are mapped to the structure and labeled red; The upstream and core motifs are based on previous ChIP-exo results. (**B**) CTCF constructs used in current study (N-terminal HALO-tag not shown); Spec-seq libraries design for unmethylated sites; R1, R2 and R3 randomize the core and upstream sites simultaneously, whereas R2-m1, m2, and m3 carry defective cores along with randomized upstream sequences; (**C**) Spec-seq data reproducibility between replicates; All observed energy values are normalized against the reference site in each sample. (**D**–**F**) Energy distribution of variants in R2 libraries with different cores are shown on the right panel, whereas the upstream motifs derived based on the corresponding cores are shown on the left panel; Part of the R567W motif that differs from wildtype are highlighted in red. (**G**) Additivity test for CTCF using four reference sites with different cores (CCGGTA, CAGGTA, CGGGTA, and CTGGTA); The observed value of double variant is plotted against the predicted value of double variant based on single variant data; RMSE were shown for each case.

Next, we explored whether other long ZFPs also show similar case of dependent recognition. CTCF, the genome insulator in the human genome, is composed of 11 tandem zinc fingers that have identical sequences between humans and mice (Figure 3A). Previous ChIP-chip and ChIP-seq work (14,29,30) identified a 14nt core motif CCNNNAGGGGGCGC recognized by fingers 7 to 3. Later Nakahashi *et al.* (21) reported extra upstream and downstream motifs with a variable 5–6 nt distance to the core (Figure 3A, downstream motif not included). According to their analysis, within 48137 detected ChIP-seq peaks, all CTCF binding sites contained features matching the core motif, but only a subset of these (∼6000 sites) contained flanking sequences matching the upstream motif adjacent to the cores. If its upstream fingers function as an independent recognition module, we expect observed ChIP-seq peaks also contain sites matching the upstream motif alone in the absence of the core motif; however, this was not observed.

To test whether CTCF’s upstream recognition is independent and how upstream specificity changes with altered core strength, we designed Spec-seq libraries (Figure 3B) in which we simultaneously altered the upstream and core sequences (R1, R2, R3). In addition, we designed three libraries (R2-m1, R2-m2 and R2-m3) to incrementally introduce more mismatches into the core and thereby test if the upstream site can still be properly recognized by fingers 9–11 in the presence of increasingly defective cores. Overall, we profiled binding of three CTCF constructs (F1–F9, F1–F11, and the known disease mutant R567W) to each library and observed good reproducibility between replicates (Figure 3C, Supplementary Figure S3).

For each construct, we then sorted results by the strength of the core and generated logos to depict the observed upstream specificity. Consistent with previous results, without fingers 10 and 11, the truncated F1–F9 construct shows no upstream specificity at all (Figure 3D), while for the F1–F11 construct, the upstream site TGCAATCCC was the optimal site associated with most cores (Figure 3E). The measured energy distribution of upstream variants varied significantly from core to core, with the strongest core CCGGT showing modest upstream specificity and cores of intermediate strength (CTGGT, CGGGT, CAGGT) exhibiting the biggest dynamic range (up to 4kT) and the strongest upstream specificity. For defective cores like GTTTTA in R2-m3, we didn’t observe any upstream motif. These results are consistent with a model in which upstream sites need only contribute a small amount of energy to form specific, stable CTCF-DNA complex in the presence of the strongest core, but even the strongest upstream site alone is insufficient to localize CTCF to a defective core. These observations cannot be explained by an additive, position-independent recognition model, under which the upstream motif profile should have no correlation with core strength at all.

We then attempted to directly quantify non-additivity via double mutant cycle analysis, in which we chose four different reference sites (CCGGT, CAGGT, CTGGT, CGGGT), perturbed the upstream and core each with single mismatches alone and in combination, and compared the observed energy of double variants to what would be predicted assuming additivity of single variant effects (Figure 3G, upper panel). While double variants at some sites contributed additively, others showed significant non-additivity, particularly when using the strong core CCGGT as reference (Figure 3G, lower panel). This observed non-additivity has practical consequences for motif finding: for example, using the weak upstream motif observed in the presence of the strong core CCGGT to predict binding energies for other sites with weaker cores (CAGGT, CTGGT, CGGGT) will systematically underestimate the true binding energy.

Besides the regular R2 library in our design, we included an ‘R2L’ library to test whether CTCF can recognize the upstream motif in extended 6nt spacing configuration, as previously reported (21). Indeed, CTCF can recognize the upstream site with the extended spacing configuration, though the observed motif appeared slightly weaker than the regular spacing case (Supplementary Figure S8).

Together, these results establish that ZFY and CTCF recognize underlying sites non-additively and have important implications for motif discovery: we cannot reliably predict the binding energy for all sites with one single PWM or PEM for long ZFPs. The use of motif or simple, additive model implicitly assumes that some configuration of high-affinity protein-DNA complex is already formed, thus the binding energy of a given sequence can be well approximated by the sum of the contribution of each base at corresponding position. However, there are many other configurations with alternate orientation or spacing like CTCF’s extended configuration (Supplementary Figure S8), albeit with lower affinity, which are not considered in this model. When some critical position (s), usually in the ‘core’ region, are mutated, the assumed configuration is severely weakened and no more stable than other configurations, thus the observed energy fall into the non-specific, plateau regime, which is the combined result of all possible configurations. Our ZFY and CTCF data showed that, all those sites with defective cores (R9, R9N, R9NN for ZFY, R2-m3 for CTCF) are in this non-specific regime (Figures 2B, 3E), regardless of the strength of flanking region. This means, due to the non-additive nature of long ZFPs, the energy decrease (affinity gain) in the flanking region cannot effectively compensate the energy increase (affinity loss) of those defective cores to form high-affinity complexes. Practically, we could not draw any specificity information from those non-specific sites (Figure 3E). In other words, each additive model has limited applicable scope.

### Upstream sites negatively regulate effects of cytosine modifications within the core

The methylation effect, or C-to-mC substitution effect, can be defined as the energy difference between methylated and unmethylated sites sharing the same sequence such that a positive effect means that methylation blocks protein-DNA interactions, known to be essential for epigenetic control by CTCF (31). Previously, our scanning of CTCF’s core site revealed that only mCpG at position 2 to 3 confers a significant energy change (25), later shown to be exclusively derived from an upper strand mC at position 2 recognized by finger 7 (32).

**Figure 4.**
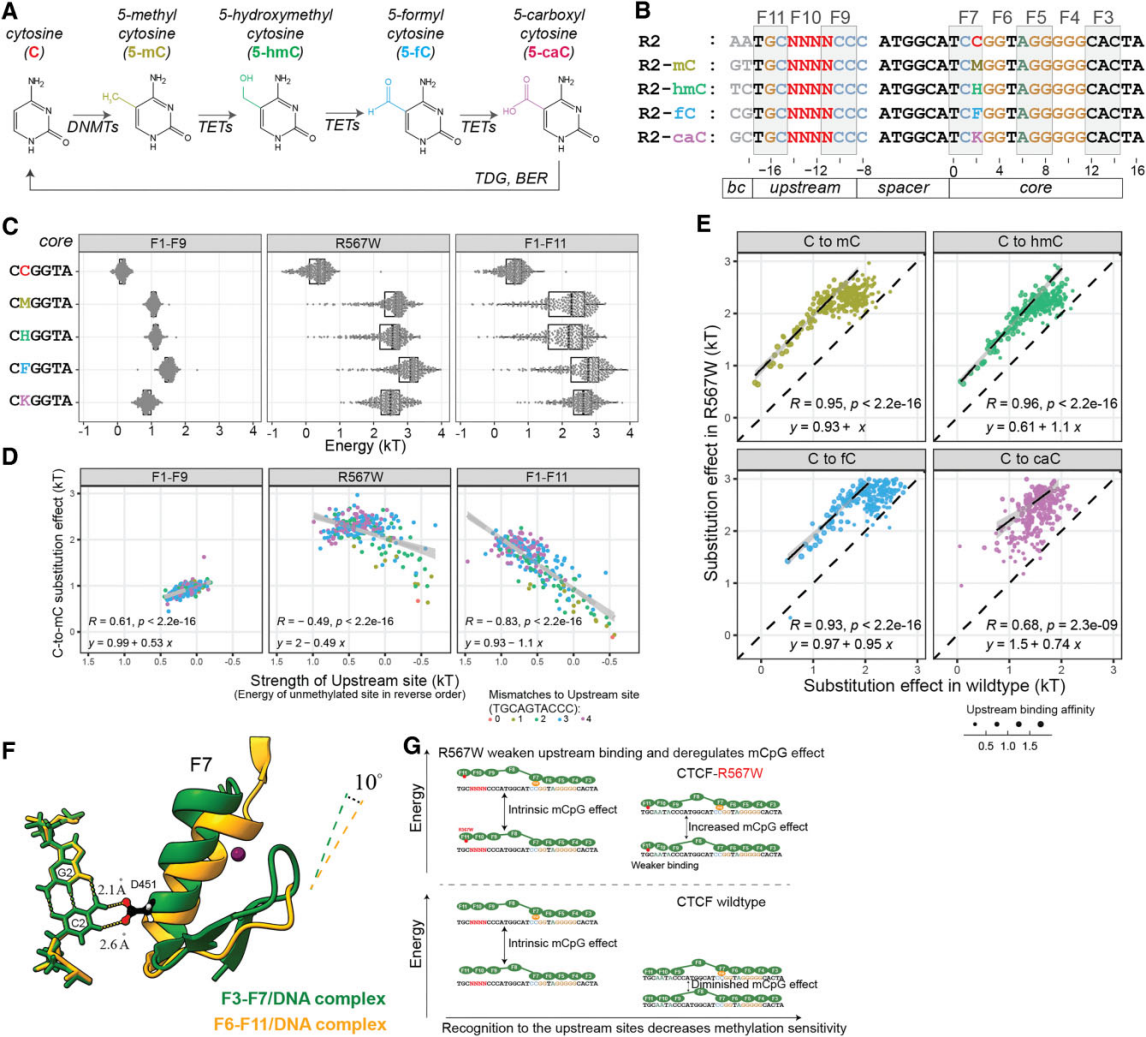
The methylation effect of CTCF recognition is negatively regulated by the strength of upstream sites. (**A**) Known cytosine methylation and modification pathways in human. (**B**) Methyl-Spec-seq libraries design testing various C-to-xC effects at position 2 with altered upstream sites. Barcodes at –19 and –18 indicate the type of modifications. (**C**) Variants distribution of binding energy with different modifications and constructs; M, H, F, K are short for methylated, hemimethylated, formyl, and carboxyl cytosines respectively. (**D**) Relationship between observed C-to-mC substitution effects and the strength of upstream site, which is defined by the energy of corresponding unmethylated site. (**E**) Comparison of C-to-xC substitution effects between wildtype and R567W mutant constructs; The dashed lines indicate linear regression of high affinity sites (with energy of unmethylated sites below 0.4kT). (**F**) Structural comparison of existing CTCF-DNA complexes; Two structures were aligned over bases C2 and G2; Only CG at position 2 and finger 7 were shown. (**G**) Proposed Upstream Regulation model of CTCF.

Besides cytosine methylation, other epigenetic marks including hydroxymethylation, formylation, and carboxylation (Figure 4A) widely exist in mammals and were suggested to have roles in CTCF binding *in vivo* (33,34). However, to date there is no systematic study of their biophysical effects upon CTCF recognition. To quantify their effects on CTCF-DNA interactions and test whether variations in the upstream site can influence the magnitude of these effects, we applied our previously developed method Methyl-Spec-seq (25). These measurements used a revised Spec-seq libraries design that included four more randomized libraries (R2-mC, R2-hmC, R2-fC, R2-caC) carrying chemically modified cytosines at the upper strand of position 2 as designated and modification-specific barcodes at positions –19 to –18 (Figure 4B).

We initially expected that any modification sufficient to disrupt finger 7 recognition would also abolish upstream site recognition, thereby enhancing the observed epigenetic effect in the presence of a strong upstream site. However, we observed several surprising results. While all tested cytosine modifications at position 2 reduced DNA binding of CTCF to different degrees (Figure 4C), TGCAATACCC remained the optimal upstream site, meaning that a single epigenetic modification is far from disruptive enough to abolish the upstream recognition. Moreover, plotting the C-to-mC effect versus the strength of the upstream site (calculated from the unmethylated case) revealed a negative correlation for the F1–F11 full-length construct but no correlation for the truncated F1–F9 construct (Figure 4D). For the optimal upstream site TGCAATACCC, the C-to-mC effect is even diminished (Supplementary Figure S9). This result was consistent across replicates and experiments and across multiple modifications (hmC, fC, and caC) (Supplementary Figure S9).

Currently the C-to-mC effect of CTCF is thought to be caused by the steric clash of aspartate residue 451 (D451) with the methylated cytosine at position 2 (C2) (32). While there is not yet a full-length CTCF-DNA crystal structure, we investigated the structural relationship between CTCF and the DNA interface by aligning partially overlapping CTCF-DNA structures as surrogates (PDB #5KKQ and #5YEL) based on the C2-G2 nucleotides and comparing their orientation difference (Figure 4F). These aligned structures show that the recognition helix of finger 7 is tilted 10 degrees away from C2 in the presence of upstream recognition, prompting us to propose a ‘Grip-and-Control’ model (Figure 4G) in which fingers 9–11 properly ‘grip’ the upstream sites and form a specific complex, leading to a conformational shift that yields a CTCF-DNA complex that is more tolerant to internal mismatches and chemical modifications, thereby decreasing the observed C-to-mC effect. Note that the carboxyl group is bulkier, rendering its effect under upstream control weaker than other modifications (Supplementary Figure S6).

From a functional perspective, this means that the epigenetic effect of methylating each CTCF site in the human genome is quantitatively fine-tuned by the upstream sequence to the desired level (0–2 kT), which could be important to fulfill the regulatory function of CTCF.

### Mutant R567W in finger 11 weakens upstream recognition and deregulates the methylation effect to the core

Next, we constructed a full-length CTCF structural model by aligning published structures (32,35), and mapping all currently identified disease mutants (36) onto this model (Figure 3A). Remarkably, all missense mutations are located within base-touching fingers (Figure 3A). R567W is particularly interesting, as it is the first identified missense mutant, originally found to cause intellectual disability, microcephaly, and growth retardation (22,23), and was later found in endometrial endometrioid adenocarcinoma, endometrial mixed adenocarcinoma, and lung adenocarcinoma (37). While R567W’s location in finger 11’s recognition helix opposing the base-contacting side suggested this mutant should only marginally affect upstream recognition while having no influence over the core, its surprising association with dementia more severe than that associated with nonsense and other missense mutants (22,38) led us to characterize it more deeply.

Methyl-Spec-seq results for R567W showed two differences from wildtype CTCF. First, R567W altered upstream specificity mainly in the R1 region (position –17 to –15, Figure 3F), altering the preferred motif from TGC to TtC. The existing structure shows arginine 567 loosely associated with the phosphate backbone, suggesting that mutating this residue could weaken the local recognition even without directly touching the base. Second, if we view the upstream fingers as a regulatory module, weakening this module could deregulate and increase the C-to-mC effect to the core. For all binding sites tested in our libraries (including high affinity upstream sequences and all different methyl modifications), the modification effect at C2 for R567W was almost always larger than for the WT case (Figure 4E).*In vivo*, we expect that the disruption of the upstream finger would selectively weaken CTCF sites with good upstream sequences, and for some of those sites containing CpG at position 2, R567W mutant could confer higher-than-normal methylation sensitivity and lower-than-normal occupancy. This can partly explain the observed clinical severity of R567W mutation: while low CTCF occupancy due to haploinsufficiency could be compensated by higher CTCF expression levels, epigenetic defects might be more difficult to rescue. Indeed, analysis of bulk RNA-seq results of blood samples (22) from CTCF mutant carriers showed that R567W mutant exhibits distinct expression profiles from LoF patients and healthy controls (Supplementary Figure S10).

### ModeMap analysis of ChIP-exo data reveal the recognition models of ZIM3 and ZNF343

**Figure 5.**
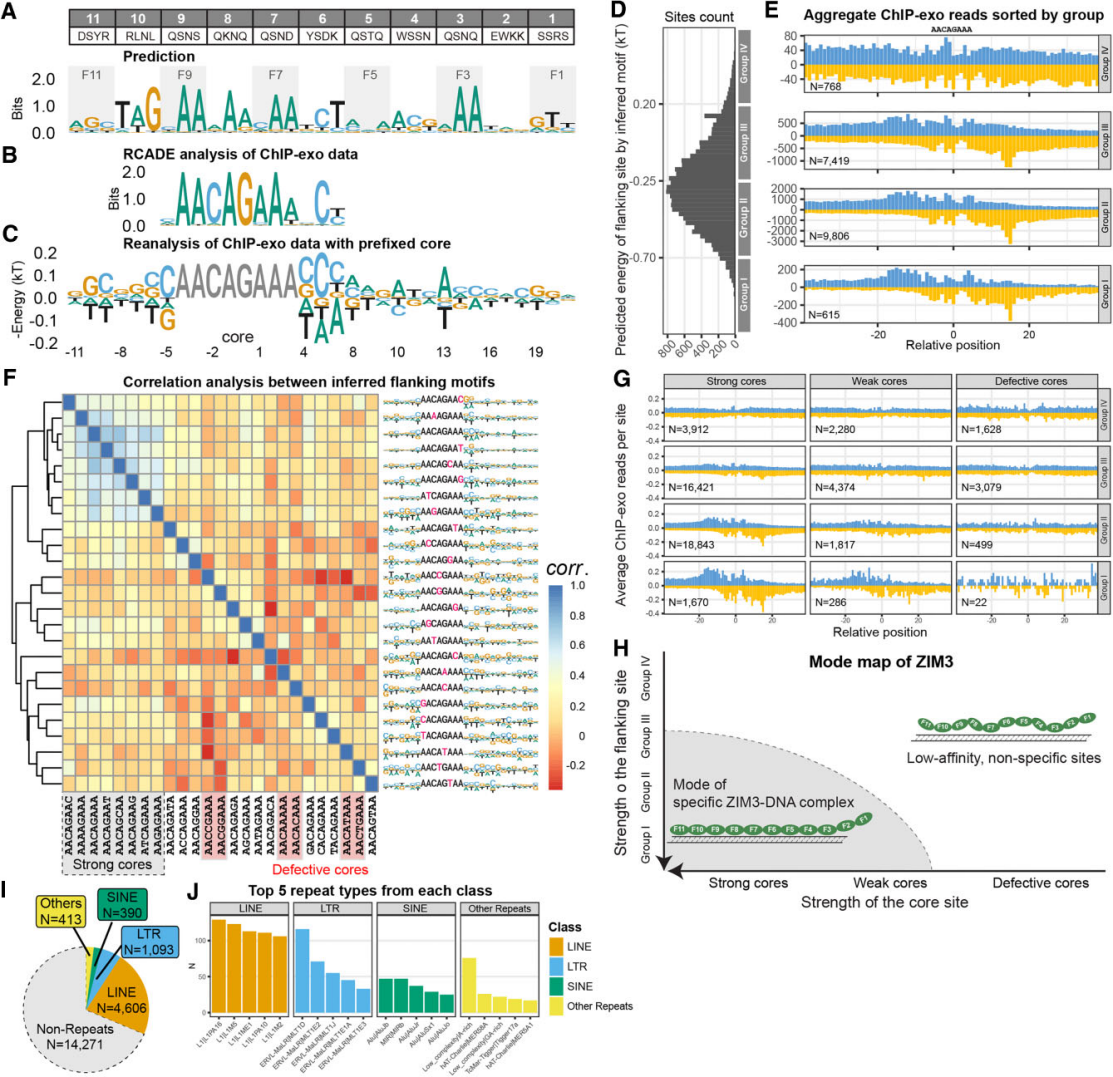
ModeMap analysis of ZIM3 reveals its extended motif, recognition model, and bound repeat elements. (**A**) Contact residues for human ZIM3; Motif prediction by B1H method. (**B**) Motif from RCADE analysis of ChIP-exo data. (**C**) Extended motif by reanalysis of ChIP-exo data with prefixed core AACAGAAA. (**D**) Distribution of binding sites according to the predicted energy of flanking sequences. (**E**) Aggregate ChIP-exo reads distribution sorted by groups with equal energy bandwidth. (**F**) Extended motifs by reanalysis of ChIP-exo data with all single variants of AACAGAAA as the prefixed core; Heatmap is generated by auto-correlation analysis of all extended motifs; Strong and defective cores are labeled with grey and red shaded boxes; remaining cores are classified as weak cores. (**G**) Re-plot of aggregate ChIP-exo signals around three classes of cores, each sorted by the flanking site strength as E) and normalized by the number of sites. (**H**) Mode map inferred from G) (**I**) Repeats distribution of *in vivo* specific sites identified within the full mode inferred from ModeMap. (**J**) Top 5 repeat types from each class among identified sites.

If the dependent recognition property revealed by ZFY and CTCF is general, current motif discovery algorithms need revision. For example, human ZIM3 is a 11-finger long KRAB-ZFP (Figure 5A), yet RCADE analysis (15) of published ChIP-exo data from H293T cells (39) only found a 10-nt long motif (AACAGAAANCT) (Figure 5B). The B1H prediction (Figure 5A) suggests that this 10-mer site is recognized by fingers 9 to 6. With this prior knowledge, we identified 18608 intact AACAGAAA sites within 38210 ChIP-exo peaks and then used these sites as a fiducial reference to align, map, and count all ChIP-exo reads based on their relative distance to the nearest anchor site (Figure 5F). Ideally, the ChIP-exo read counts near each site should be proportional to its binding occupancy; if the *in vivo* ZIM3 protein concentration is low enough, reads should also be proportional to the binding affinity. Based on these assumptions, we calculated the negative logarithmic ratio of ChIP-exo reads near each site as an estimate of its relative binding energy, and then performed data regression to infer the motifs (Figure 5C).

To cross-validate the predictive power of this inferred motif, we used it to predict the binding energy of all tested sites, sorted them into four groups with equal energy bandwidth (Figure 5D), then plotted the aggregate ChIP-exo reads around each group of sites (Figure 5E). Clear, asymmetric bi-peaks signals emerged at –12 and +15 positions of opposing strands for Group I sites, and gradually disappeared towards Group IV sites, confirming that this inferred flanking motif effectively distinguishes high affinity, specific sites from non-specific sites. In comparison, when we used the RCADE flanking motif (Figure 5B) at positions (–5, 4, 5, 6) to make the same group-wise footprinting, we cannot separate sites as well as our inferred motif (Supplementary Figure S11), strongly suggesting the extra motif learned through our analysis confer additional predictive power for specific sites.

Encouraged by these results, we applied the same fixed-core analysis to all other 24 cores with no more than one mismatch to the intact core, then performed auto-correlation analysis and hierarchical clustering based on similarity of the inferred flanking motifs to each other (Figure 5F). Less than one third of them (7 out of 25) yield flanking motifs similar to the intact core case, thus we classified them as strong cores; close inspection of the ChIP-exo footprints around each core revealed at least six cores (those with mismatches at position 0 or –1) cannot elicit characteristic bi-peaks signals, regardless of group assignment, thus they were designated as defective cores; the remaining eleven cores were classified as weak cores. For better visualizations, we remade ChIP-exo footprints upon these three classes of cores respectively, each with four groups sorted by the same flanking motif found in intact core case, and the ChIP-exo reads are normalized by count of sites in each group. Group I, II, III sites associated with strong cores exhibit clear bi-peaks signals; for weak cores, only Group I sites elicit such signals; for defective cores, no bi-peaks signals were detected at (–12, +15) positions. These results show a promising picture about the recognition model of ZIM3 (Figure 5H), i.e. there exists a mode defined by the strength of core and flanking sites together, under which ZIM3 can form a specific complex with at least eight fingers engaged in the recognition. This picture is also consistent with the dependent recognition property learned from ZFY and CTCF, i.e. it is futile to search (Figure 5F) and meaningless to use flanking motif for prediction (Supplementary Figure S13) around those defective cores, because any single mismatch at core position 0 or –1 severely destabilize the assumed specific complex, not to mention those double or triple mismatches. Note that since very few reads were detected around limited number of defective cores within ChIP-exo peaks, to increase the reliability of our model, we redid footprinting upon all possible defective cores across the human genome, and the conclusions do not change (Supplementary Figure S12).

ZIM3 is not unique. When we applied above procedures to ZNF343, another 12-finger long KRAB-ZFP, the inferred flanking motif is highly consistent with published HT-SELEX result and only 4 out of 19 hexamer cores support the formation of long, specific complexes (Supplementary Figure S14). To facilitate broad adoption by other labs to study other long ZFPs, we named the above analysis workflow ModeMap, and deposited data and codes under public repository (Supplemental Table S3).

### Repeats-derived elements can be reliably annotated through ModeMap

Previous annotations of KRAB-ZFP’s association with repeat elements are usually based on the overlap of top ChIP-seq peaks with repeats within the genome (15,39), which could cause ambiguity, particularly for those short repeats like Alu elements. Through ModeMap, we clearly know the conditions to form specific ZFP-DNA complex. For ZIM3, if we conservatively define specific sites to be Group I, II sites associated with strong cores and Group I sites associated with weak cores, then one third of ZIM3’s specific sites are located within some repeat elements called by RepeatMasker, mostly L1 family elements (Figure 5I). If the primary function of ZIM3 is to target and silence L1 repeats, we expect ZIM3 motif closely matches the consensus sequence of the targeted position of corresponding repeat, so as many repeat instances as possible can be bound. In fact, it binds many diverse positions within each repeat type and our inferred motif doesn’t match the underlying consensus sites very well (Supplementary Figure S15). Probably, ZIM3 has some endogenous function we don’t know yet, and these repeats-derived sites contributed to the evolution of ZIM3’s regulatory networks, not vice versa.

## DISCUSSION

We think that there are two technical issues and a biophysical reason that explain the current ‘Long fingers but short motifs’ conundrum. First, most existing techniques like PBM, ChIP-seq, and Affinity-seq have limited power to resolve long binding sites. For ChIP-seq measurements on human genomic DNA, each 15-mer show up only once on average, making the discovery of longer motifs increasingly difficult. Second, the irregular nature of extended motifs like those seen for ZFY, where each finger does not appear to interact with the ‘expected’ three bases (40), can also inhibit motif detection when searches are guided by motif prediction methods. Lastly and importantly, the core motif often specifies binding above some baseline threshold, and in its absence, the flanking region doesn’t support the formation of high-affinity complex, though the extended motif does contribute to recognition in the context of ‘good’ core. Without knowing that, we would search for motifs around those defective cores, which is futile and interfering. To improve this situation, we developed ModeMap to search motifs around ‘good’ cores first and then infer the conditions to form the assumed specific complexes. Either the ChIP-exo footprints or HT-SELEX results can cross-validate our ModeMap predictions well.

Besides impeding motif discovery, the dependency effect implies the functions of those ‘extra’ fingers are underestimated. For CTCF, we showed that its secondary fingers can modulate the specificity and methylation sensitivity to the core by tuning the strength of upstream sites, essentially serving as an ‘epigenetic modulator’. For ZFY, though its downstream fingers have limited contribution to overall target recognition, they still significantly increase the residence time on binding sites accommodating more fingers, which could be important to fulfill ZFY’s biological function. For many more ZNFs, it is likely that ChIP-derived motifs are the minimal core motifs needed for binding above baseline levels and thus can explain most of genomic binding events well, but higher affinity and longer residence time towards sites matching full-length motifs are needed to fulfill their functions (41), such as silencing transposable elements by KRAB-ZNFs. Further *in vivo* experiments are required to validate the significance and generality of these findings.

This work is one of a series of work about the form, mode, functions, and diseases mechanism of ZFPs. Majority of long ZFPs contain tandem fingers organized into one closely-packed array, but in some cases, e.g. ZFP57, MECOM and PRDM16, their fingers are organized in the form of separate sub-arrayss. Probably, they have alternate recognition modes beyond currently studied cases to serve their functions. With revised concept, technique, and algorithm, we can decipher their broader roles in human biology and diseases.

## DATA AVAILABILITY

Supplemental information includes all nucleotide sequences, experimental conditions and procedures, anisotropy measurement descriptions and record. All raw and processed sequencing data used in this study are available through NCBI GEO database (GSE111772, GSE109098, GSE188164). The software packages TFCookbook, TECookbook and data analysis workflow are available through DOI: https://doi.org/10.5281/zenodo.7711887, https://doi.org/10.5281/zenodo.7711911 and https://doi.org/10.5281/zenodo.7711894, respectively.

## Supplementary Material

gkad207_Supplemental_File
