# Supplementary material for: On the dependent recognition of some long zinc finger proteins
Source: Nucleic Acids Res. 2023 Mar 23;51(11):5364–76. doi: 10.1093/nar/gkad207 (PMC10287918; doi:10.1093/nar/gkad207)
Supplement: gkad207_Supplemental_File [file gkad207_supplemental_file.pdf]

Supplemental Information and Experimental methods descriptions

Protein constructs and expression

For ZFY-related proteins, 6x-His-SUMO tag was fused to the N-terminus of ZFP coding sequences and cloned into NEB DHFR construct, whereas for CTCF and its mutant, 6x-His-HALO tag was fused to the N-terminus of CTCF coding sequences with TEV protease cleavage site as linker sequence as Fig. S1 and S2.

Protein (Uniprot ID)	Construct	Included coding region
Human ZFY (P08048)	hisSUMO-ZFY-full	390-782
	hisSUMO-ZFY(F11-F13)	710-768
	hisSUMO-ZFY(F9-F13)	653-768
	hisSUMO-ZFY(F7-F13)	596-768
	hisSUMO-ZFY(F5-F13)	539-768
	hisSUMO-ZFY(F1-F11)	408-744
Mouse ZFY1 (P10925)	hisSUMO-mZFY1(F7-F13)	578-782
CTCF (Q61164-1)	hisHALO-CTCF(F1-F9)	241-523
	hisHALO-CTCF(F1-F11)	241-583
	hisHALO-CTCF(F1-F11)-R567W	241-583

Table S1, recombinant proteins used in current work

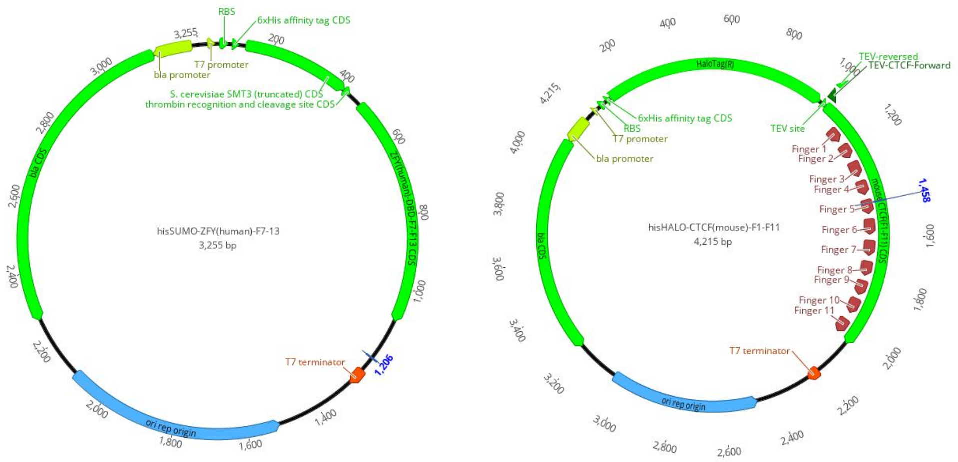

Figure S1 The ZFY and CTCF constructs used in current study.

Spec-seq libraries sequences, and experimental procedures

For ZFY:

```
ZFY-Rand1:   GATAGTCTCATTTTCACCA NNNNTAGGCGTTTCGC AGATCGGAAGAGCACACG
ZFY-Rand2:   GATAGTCTCATTTTCACCA GGCCNNNNCGTTTCGC AGATCGGAAGAGCACACG
ZFY-Rand3:   GATAGTCTCATTTTCACCA GGCCTAGGNNNNTCGC AGATCGGAAGAGCACACG
ZFY-Rand4:   GATAGTCTCATTTTCACCA GGCCTAGGCGTTNNNN AGATCGGAAGAGCACACG
ZFY-Rand5:   GATAGTCTCATTTTCACCT NNNNTAGGCGTTTTTG AGATCGGAAGAGCACACG
ZFY-Rand5N:  GATAGTCTCATTTTCACCT NNNNTTATGATTTTTG AGATCGGAAGAGCACACG
ZFY-Rand6:   GATAGTCTCATTTTCACCT GGCCNNNNCGTTTTTG AGATCGGAAGAGCACACG
ZFY-Rand7:   GATAGTCTCATTTTCACCT GGCCTAGGNNNNTTTG AGATCGGAAGAGCACACG
ZFY-Rand8:   GATAGTCTCATTTTCACCT GGCCTAGGCGTTNNNN AGATCGGAAGAGCACACG
```

```
ZFY-Rand9:    GATAGTCTCATTTTCACCT NNNNTAGTCGTTTTTG AGATCGGAAGAGCACACG
ZFY-Rand9N:   GATAGTCTCATTTTCACCT NNNNTCACGATTTTTG AGATCGGAAGAGCACACG
ZFY-Rand9NN:  GATAGTCTCATTTTCACCT NNNNTCACGATTGCCC AGATCGGAAGAGCACACG
ZFY-Rand10:   GATAGTCTCATTTTCACCT GGCCNNNNCGTTTTTG AGATCGGAAGAGCACACG
ZFY-Rand11:   GATAGTCTCATTTTCACCT GGCCTAGTNNNNTTTG AGATCGGAAGAGCACACG
ZFY-Rand12:   GATAGTCTCATTTTCACCT GGCCTAGTCGTTNNNN AGATCGGAAGAGCACACG
```

For CTCF:

```
CTCF-R1:     CGTGTGCTCTTCCGATCT AA NNNAGTGCCCATGGCATCNGGTAGGGGGCACTATCGAGAT
CTCF-R2:     CGTGTGCTCTTCCGATCT AA TGCNNNNCCCATGGCATCNGGTAGGGGGCACTATCGAGAT
CTCF-R3:     CGTGTGCTCTTCCGATCT AA TGCAGTGNNNATGGCATCNGGTAGGGGGCACTATCGAGAT

CTCF-R2L:    CGTGTGCTCTTCCGATCT AT GCNNNNCCCAGTGGCATCCGGTAGGGGGCACTATCGAGAT

CTCF-R2-m1: CGTGTGCTCTTCCGATCT AA TGCNNNNCCCATGGCATCTTGTAGGGGGCACTATCGAGAT
CTCF-R2-m2: CGTGTGCTCTTCCGATCT AA TGCNNNNCCCATGGCATGTTGTAGGGGGCACTATCGAGAT
CTCF-R2-m3: CGTGTGCTCTTCCGATCT AA TGCNNNNCCCATGGCATGTTTTAGGGGGCACTATCGAGAT

CTCF-R2-mC :CGTGTGCTCTTCCGATCT GT TGCNNNNCCCATGGCATCMGGTAGGGGGCACTATCGAGAT
CTCF-R2-hmC:CGTGTGCTCTTCCGATCT TC TGCNNNNCCCATGGCATCHGGTAGGGGGCACTATCGAGAT
CTCF-R2-fC :CGTGTGCTCTTCCGATCT CG TGCNNNNCCCATGGCATCFGGTAGGGGGCACTATCGAGAT
CTCF-R2-caC:CGTGTGCTCTTCCGATCT GC TGCNNNNCCCATGGCATCKGGTAGGGGGCACTATCGAGAT
```

M,H,F,K are short for methylated, hemimethylated, formyl, and carboxylated cytosines respectively; Randomized region are labeled red; Modification-specific barcodes are labeled green.

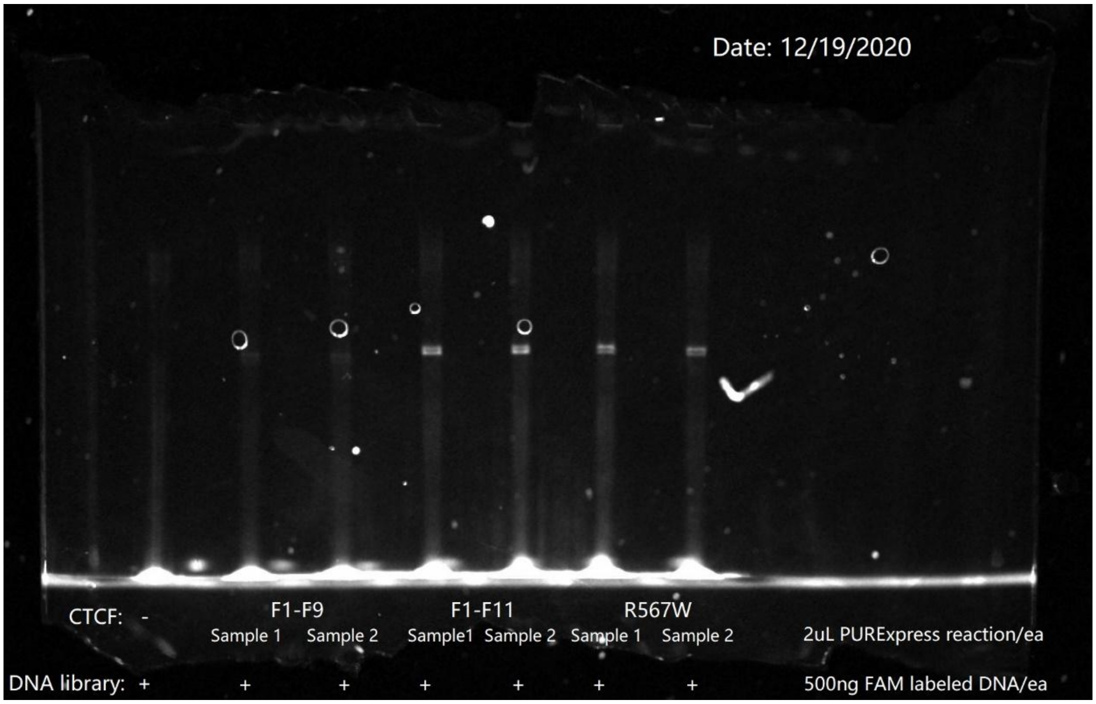

Figure S2 EMSA separation of bound CTCF-DNA complexes from unbound DNA in various constructs. The binding reaction volume for each sample is 20uL, added with 2uL PURExpress reaction containing CTCF construct. Before sample loading, all reactions are equilibrated at room temp for at least 30mins. 12% Tris-glycine gels were loaded with samples at cold room. Running conditions were set at 200V, 50mins.

Data analysis procedures and reproducibility check

The data analysis protocol for ZFY and CTCF are very similar to previous work. General introduction to the data analysis protocol can be found at https://github.com/zeropin/ZFPCookbook.

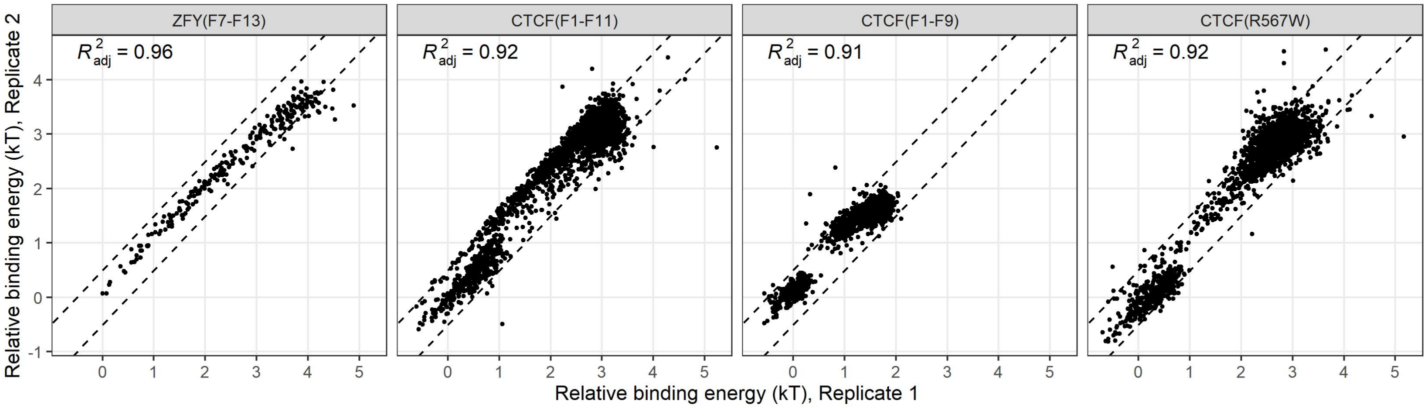

Figure S3 Data reproducibility for different constructs (Dashed lines are 0.5kT energy deviation bounds).

Supplemental Informaion for ZFY results

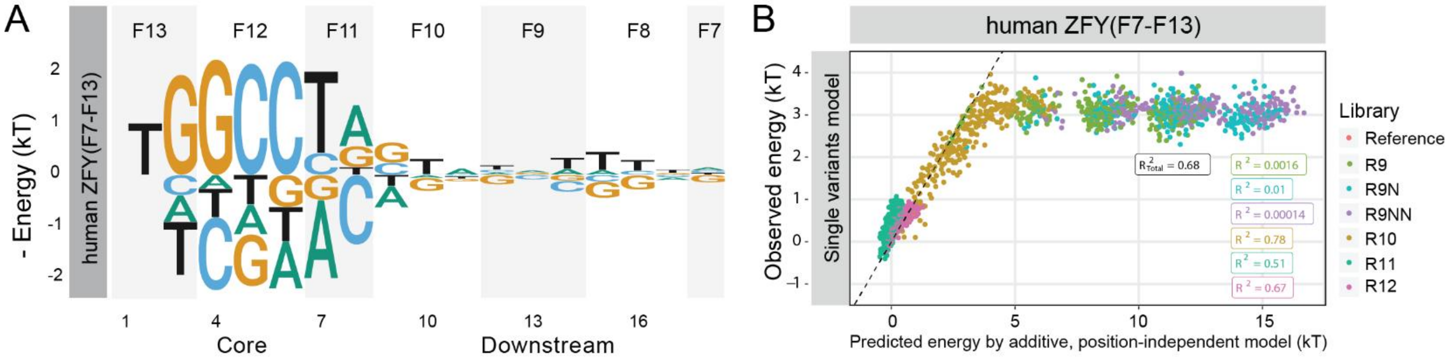

Figure S4 A) Motif logo of ZFY(F7-F13) generated by regression of energy values of all single variants of reference site; B) Comparison of Observed binding energy values with predicted values by single variants model.

Fluorescence anisotropy

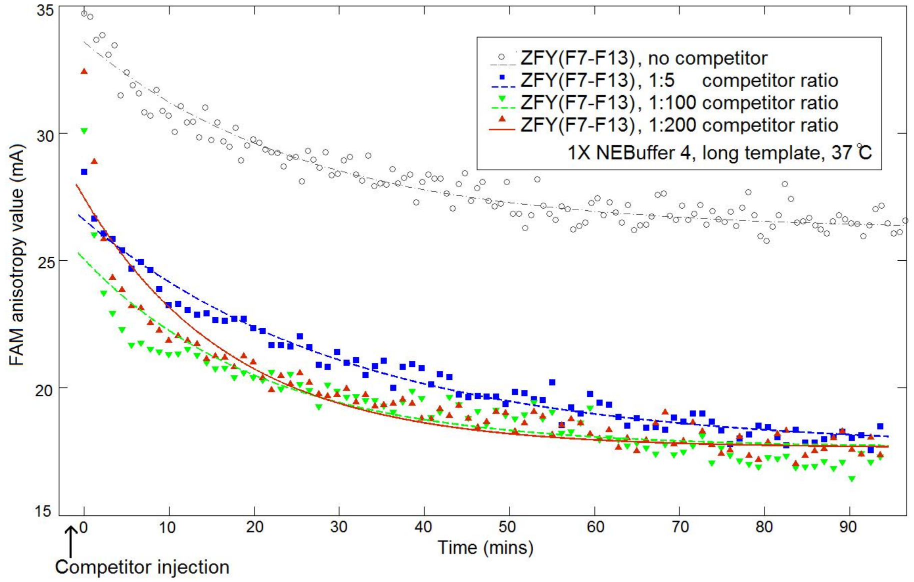

Figure S5. Titration effects of unlabeled competitor DNA on FAM-labeled probe DNA. For ZFY(F7-F13), with everything else being the same, different amount of competitor probe were added into binding reaction and the FAM anisotropy values were monitored over time. 200 molar excess of unlabeled competitor probe was found to be more than enough to prevent reassociation of ZFY-DNA complex and thus chosen for dissociation assay.

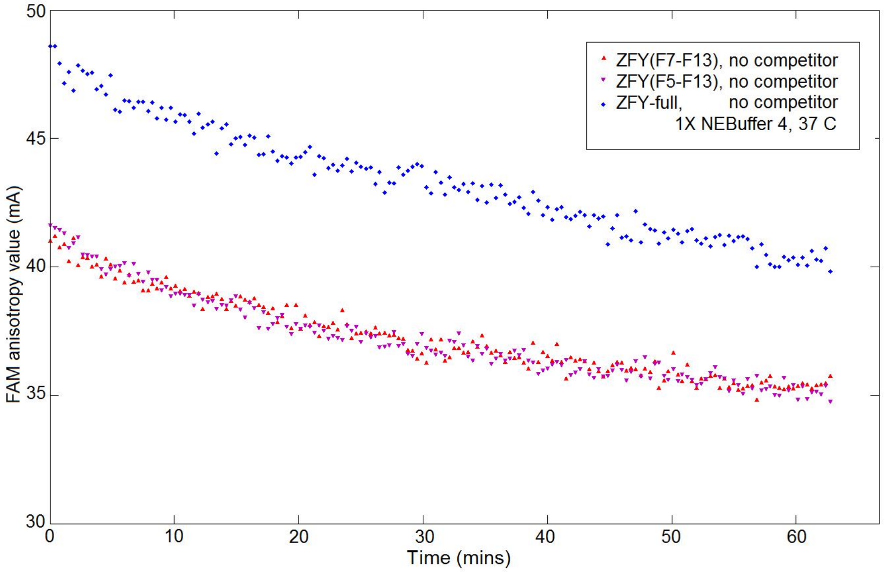

Figure S6. Comparison of inactivation rates without competitor DNA. Without competitor probes, we still observed slowly decreasing anisotropy values over time, most likely due to spontaneous inactivation of the zinc finger proteins in room temperature. The observed inactivation rates showed no major difference between different constructs and are significantly slower than the measured dissociation rates.

DNA	Protein	#1	#2	#3	#4	#5	#6	#7	Average(s)	Standard

template	construct									deviation (s)
ZFY-reference-long	ZFY(F9-F13)	1049	1103	1075	921	1142			1058	84
ZFY-reference-long	ZFY(F7-F13)	1245	1410	1272	1447	1433	1258	1443	1358	95
ZFY-reference-long	ZFY(F5-F13)	1835	1756	2003					1865	126
ZFY-reference-long	ZFY-full	2590	3127	2571					2763	316
ZFY-reference-short	ZFY-full	1176	1007	1090	993				1067	85
ZFY-reference-short(weak)	ZFY-full	656	726	609					664	59
Non-specific site (ZFP57 reference site)	ZFY-full	191	117	106					138	46

Table S2. Measurement results for dissociation kinetics assay. All measurement results are based on fitting the observed FAM anisotropy vs. time values to single phase exponential curves and the mean lifetimes were reported for each decay curves.

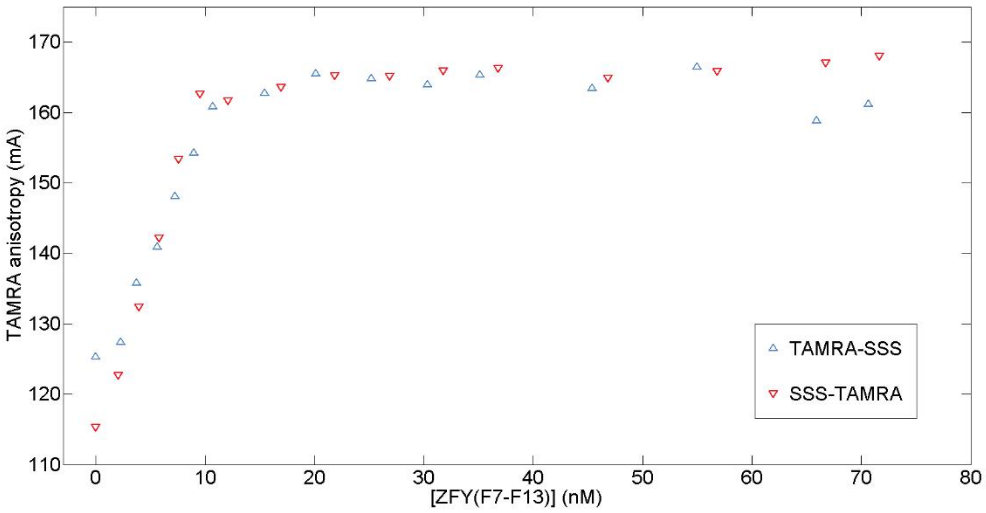

Figure S7. TAMRA anisotropy measurement of ZFY(F7-F13) dissociation constants to S-S-S probes. 5nM DNA probes are used in 1X NEBuffer 4, room temperature binding reactions. Since the Kd are comparable to the probe concentrations, there exist some uncertainty in accurate determination of Kd, but it was estimated to be around 5nM. Protein concentrations are calibrated to BSA standard by SDA-PAGE gel staining.

Upstream motifs of CTCF in regular and extended spacing formats

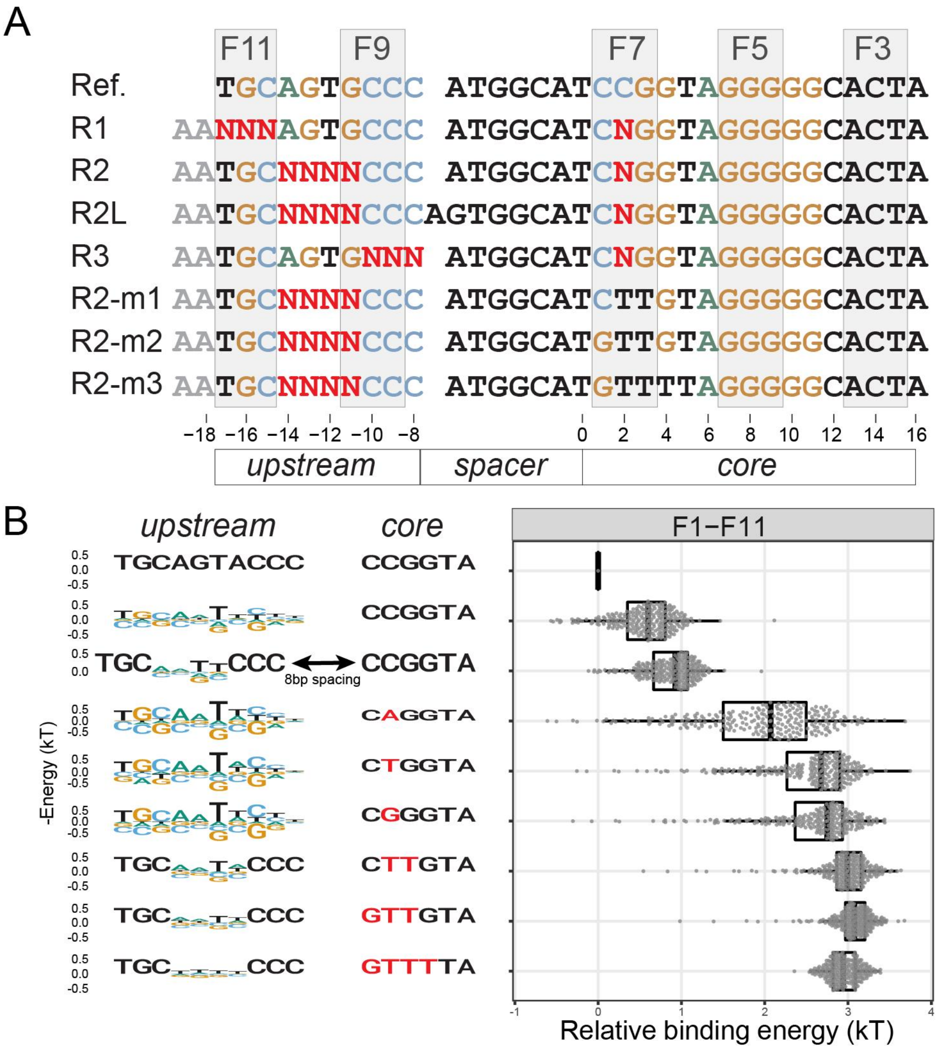

Figure S8 A) Spec-seq libraries for CTCF, including R2L with extended spacing format; B) Upstream motif profiles associated with different cores, including extended spacing format R2L library.

Methylation effects on CTCF-DNA interactions

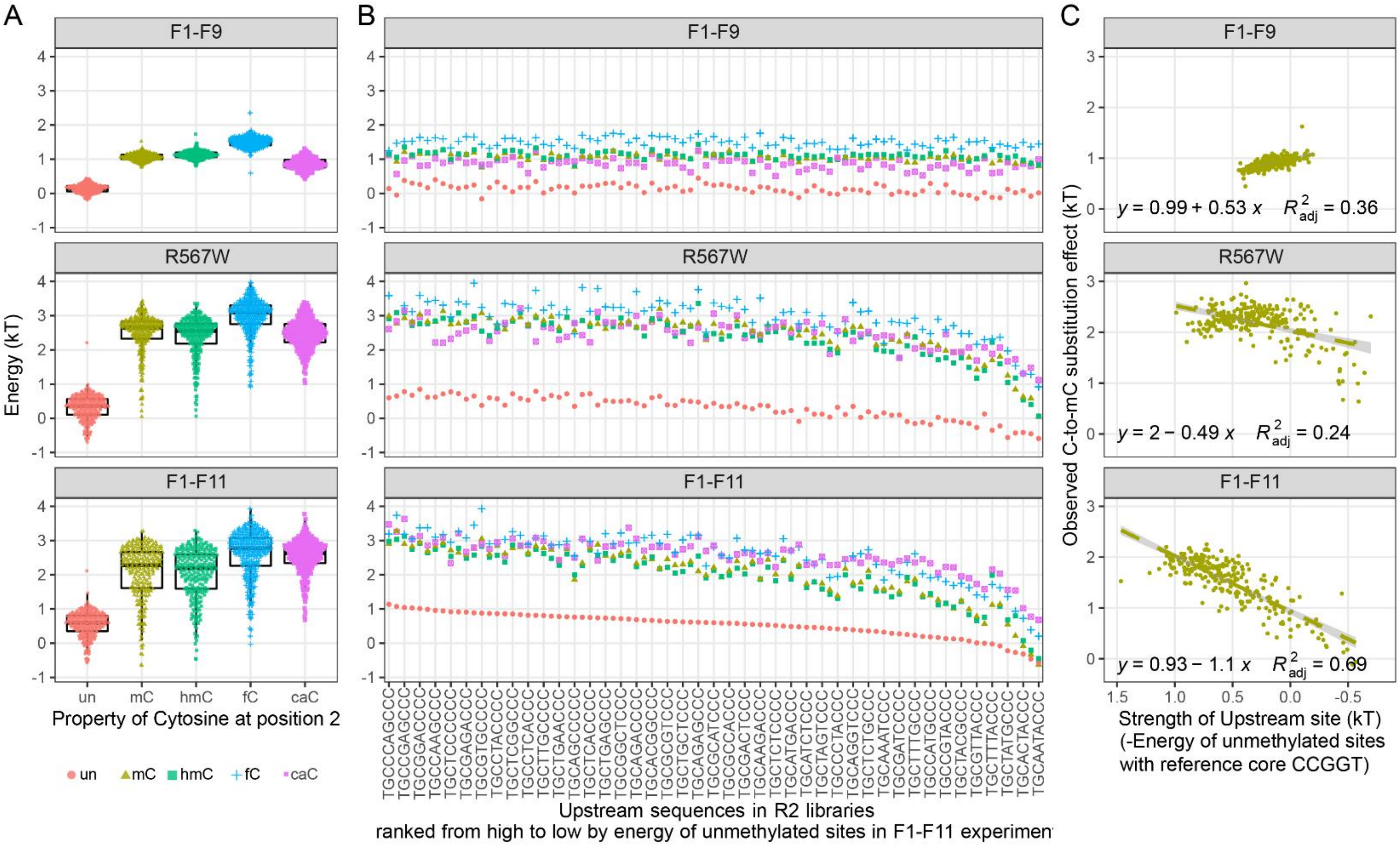

Figure S9 Methylation effects over different DNA sequences by CTCF constructs.

A) The energy distribution of different variants by types of modifications and constructs;

B) Different types of variants are ranked from low to high affinity by strength of the upstream sites;

C) Relationship between observed C-to-mC substitution effect with the strength of upstream sites.

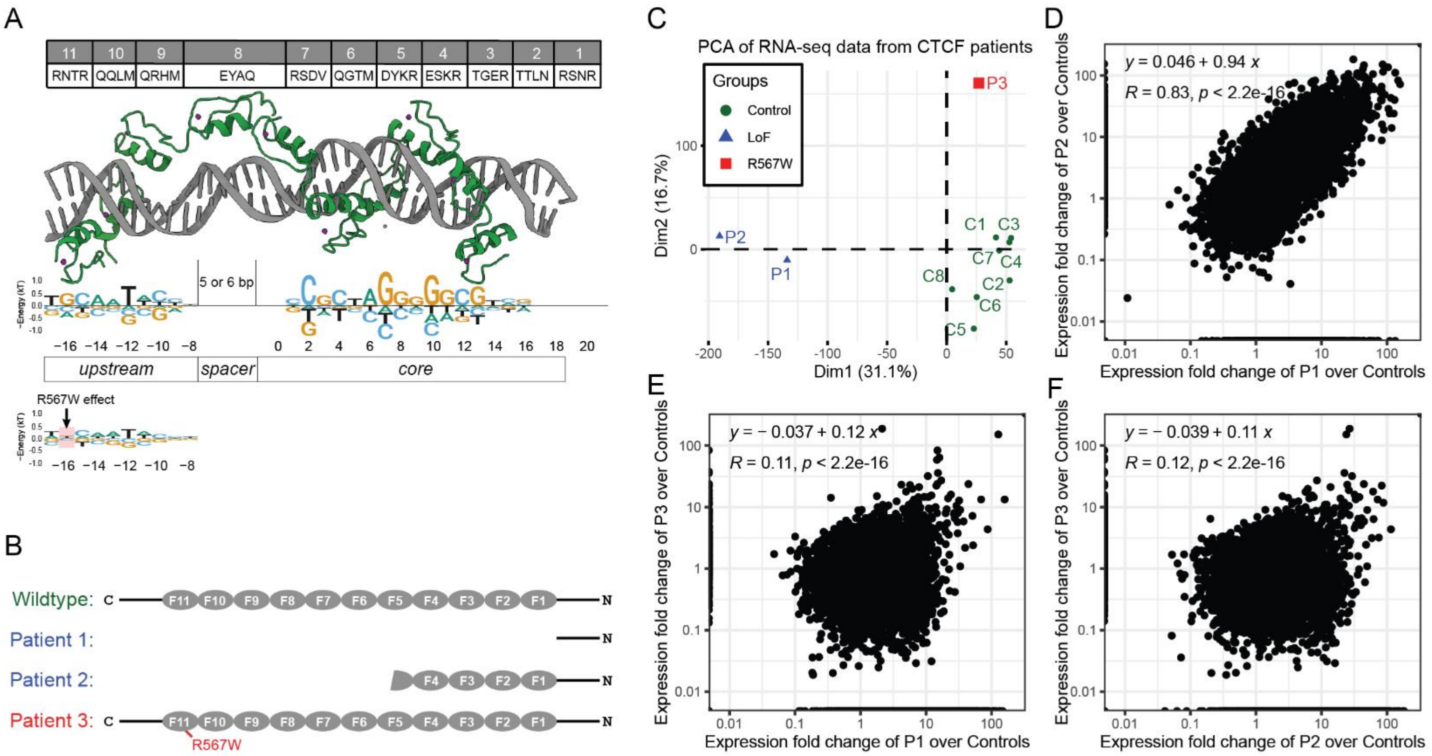

Figure S10 RNA-seq analysis of blood samples from CTCF R567W patients and LoF patients.

A) Structural model of CTCF recognition;
B) CTCF mutation maps from current identified patients;
C) PCA of RNA-seq data from CTCF patients and healthy controls;
D) Comparison of expression change between patient 2 to patient 1;
E) Comparison of expression change between patient 3 to patient 1;
F) Comparison of expression change between patient 3 to patient 2.

Supplemental Information for ZIM3 and ZNF343 results

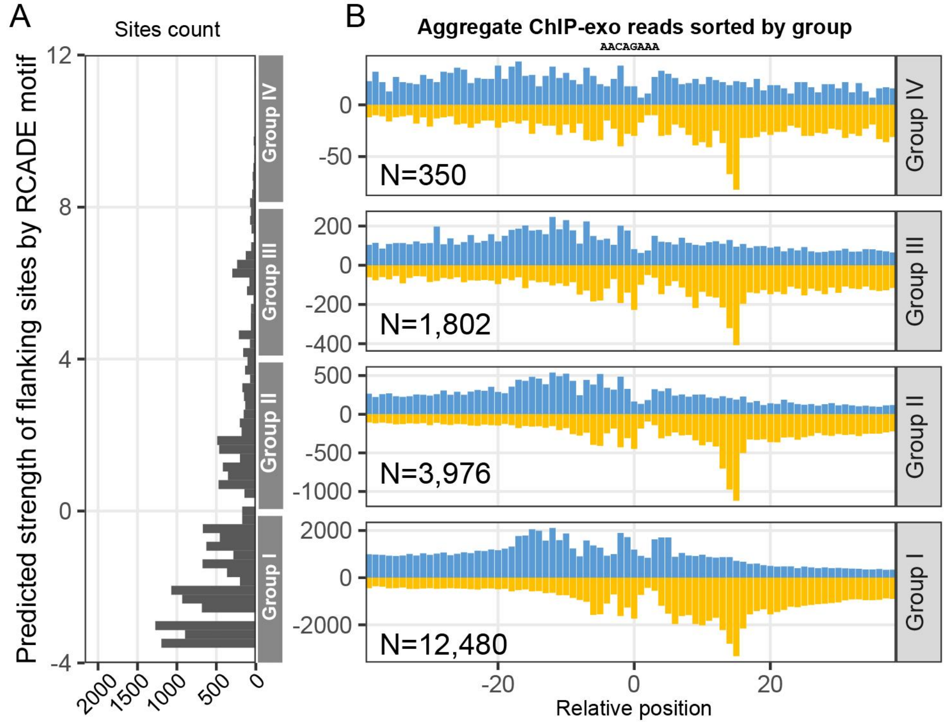

Figure S11. Distribution of binding sites strength estimated by RCADE's analysis of ZIM3's flanking motifs at positions (-5, 4, 5, 6) and the aggregate ChIP-exo footprinting plots as in Fig. 5E.

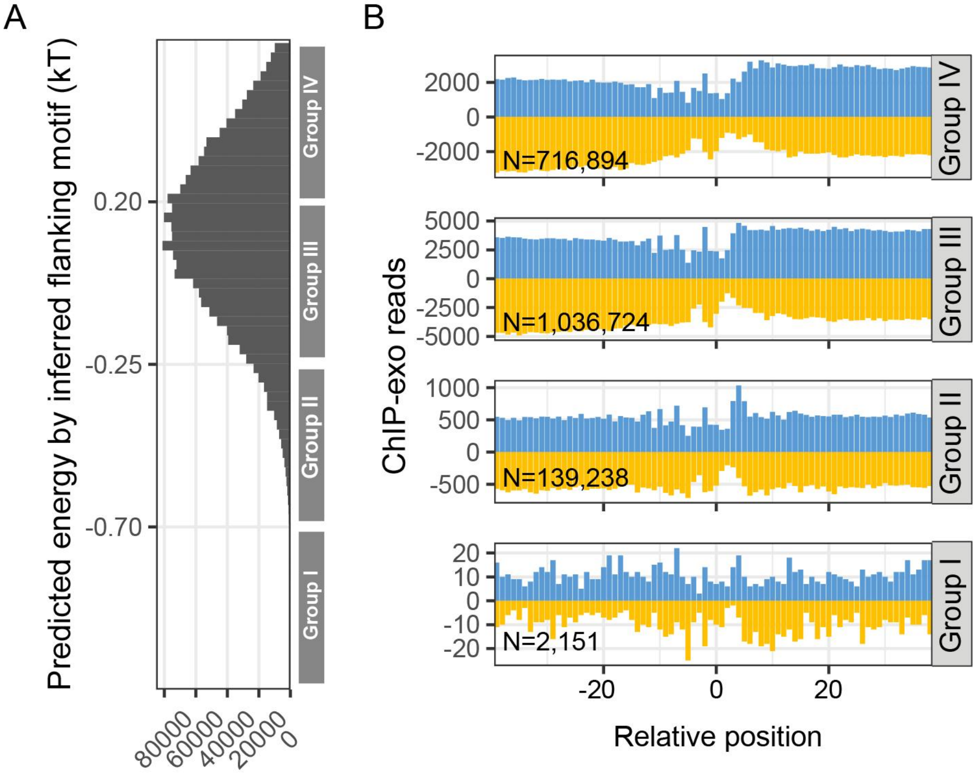

Figure S12. Aggregate ChIP-exo footprinting of all possible defective core sites within human genome

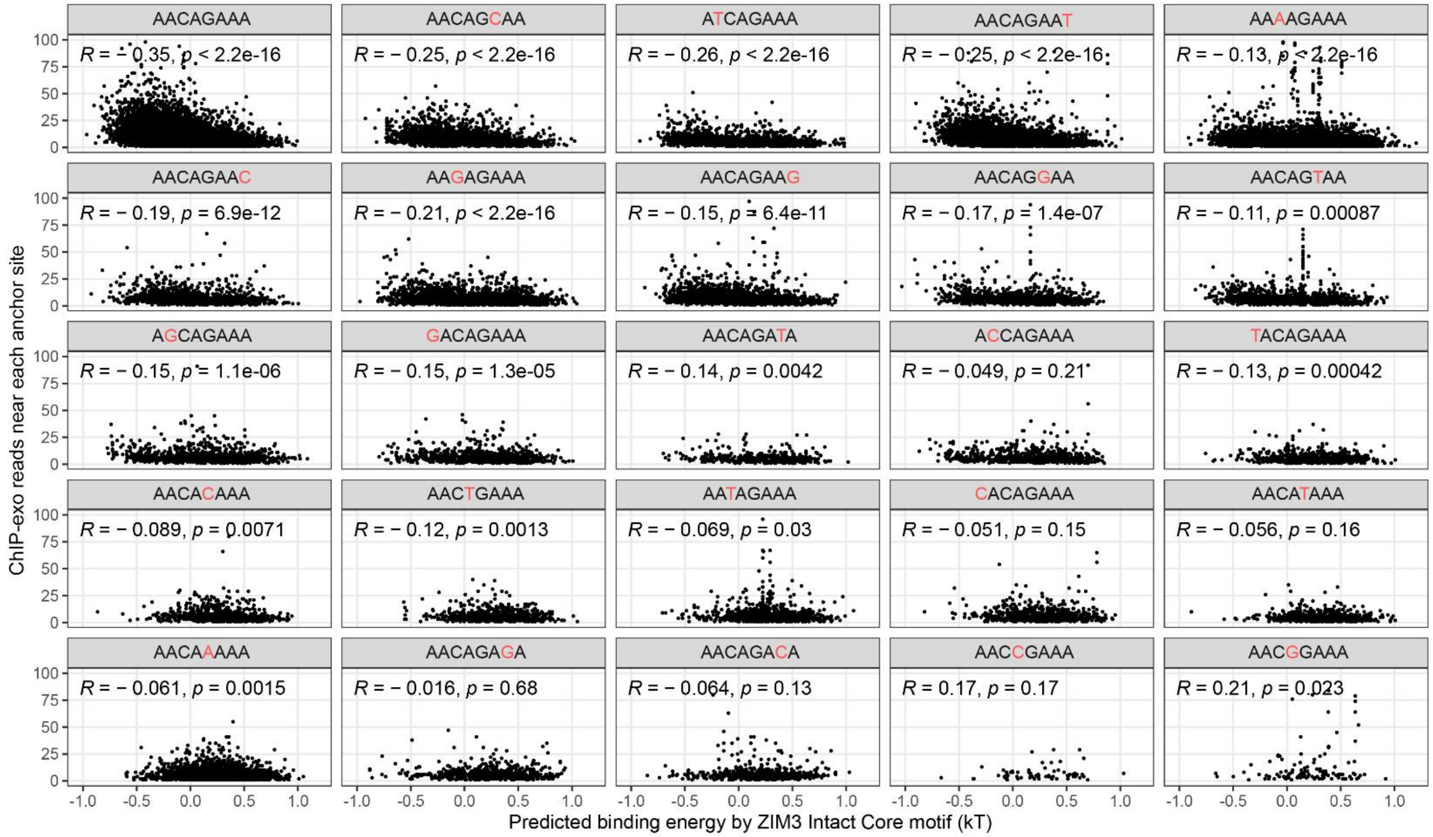

Figure S13. Correlation analysis between ChIP-exo reads around each type of anchor site and predicted binding energy by inferred ZIM3 motif from intact core case.

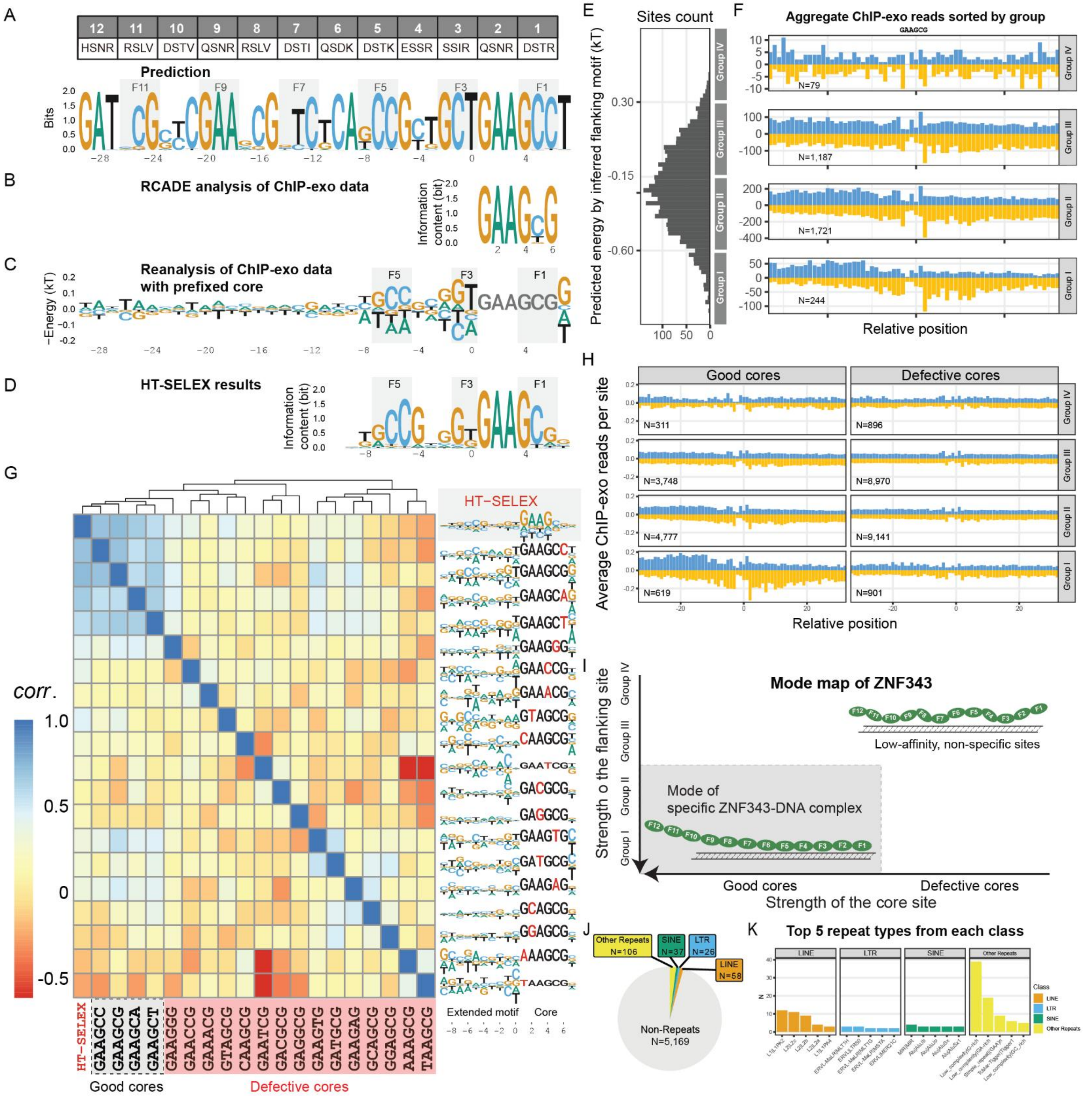

Figure S14

A) Contact residues for human ZNF343; Motif prediction by B1H method;

B) Motif from RCADE analysis of ChIP-exo data;

C) Extended motif by reanalysis of ChIP-exo data with prefixed core GAAGCG;

D) HT-SELEX results of ZNF343;

E) Distribution of binding sites based on predicted binding energy by inferred flanking motifs;
Sites can be further sorted into four groups with equal energy bandwidth;

F) Aggregate ChIP-exo reads by Group, with GAAGCG prefixed in -3 to +2 positions;

G) Extended motifs by reanalysis of ChIP-exo data with all single variants of GAAGCG as the prefixed core;
Heatmap is generated by auto-correlation analysis of all extended motifs with different cores and HT-SELEX result; The ChIP-exo reads footprints near associated prefixed cores are shown on the right;

H) Aggregate ChIP-exo signals classified by type of cores and groups, respectively; The reads number are normalized by number of sites within each group;

I) Inferred recognition model of ZNF343;

J) Annotation of identified specific binding sites in Group I, II associated with good cores;

K) Top five repeat names for each repeat classes among specific binding sites.

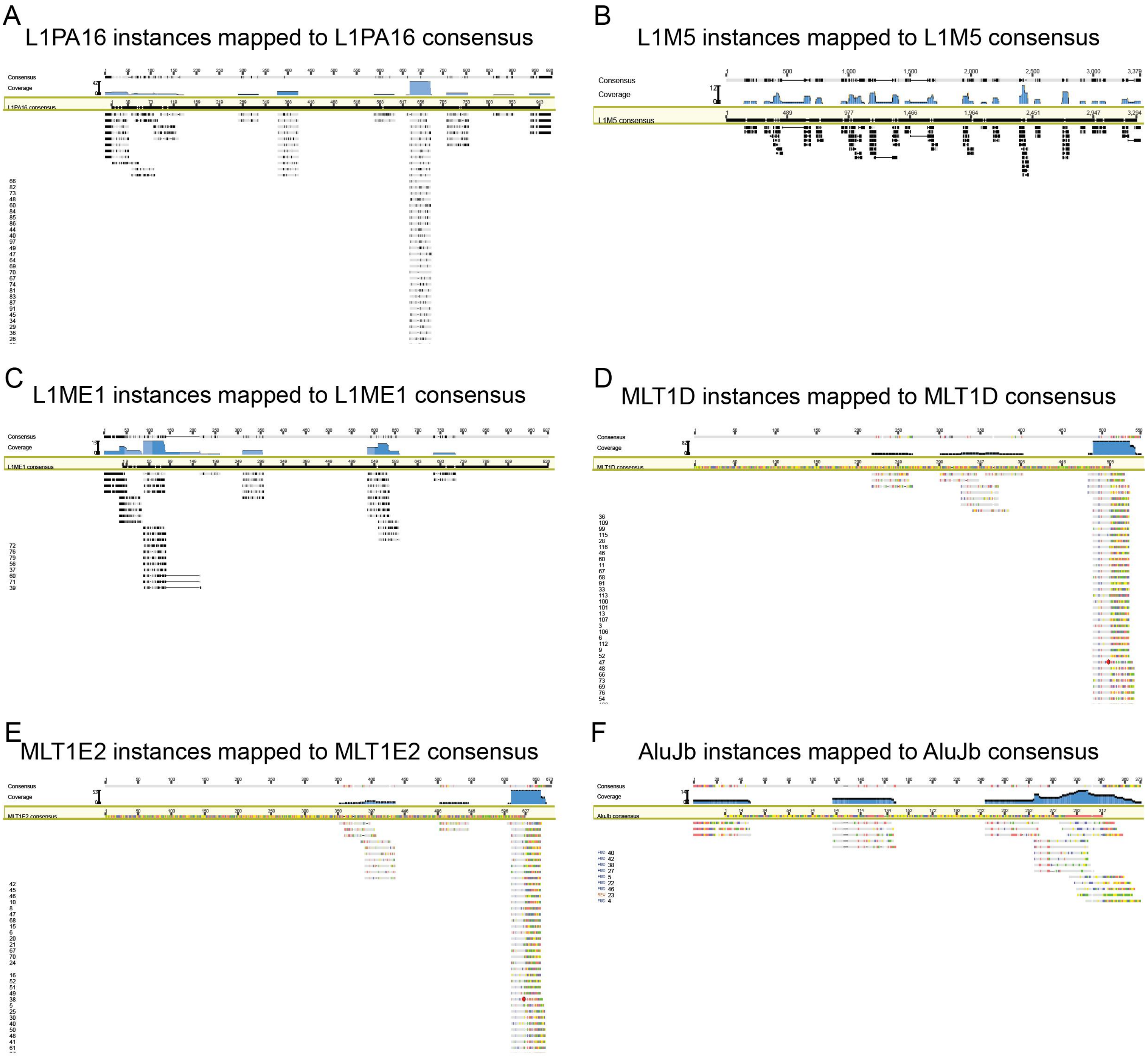

G

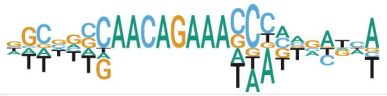

Class	Family	Name	Consensus sequence in targeted positions
LINE	L1	L1PA16	ACGCAGGAACAGAAAACCAAATACC
LINE	L1	L1M5	TCAATGGAACAGAATAGAGAGTCCA
LINE	L1	L1ME1	ATAAGAAAACAAACAACCCAATTAA
LTR	ERVL-MaLR	MLT1D	CAGCAGCAATAGGAAACTAATACA
LTR	ERVL-MaLR	MLT1E2	CGGCAGCAATAGAAAACTAATACA
SINE	Alu	AluJb	CCTGGGCGACAGAGCGAGACCCTGT

A-F) Identified repeats elements from ZIM3's specific binding sites mapped to the consensus sequence of each repeat type;
G) The inferred motif of ZIM3 in comparison of targeted positions within each repeat.

Figure S15. Alignment of identified ZIM3 specific binding sites mapped to the consensus sequence of corresponding repeat element.

REAGENT or RESOURCE	SOURCE	ACCESS IDENTIFIER
**Bacterial and Virus Strains**		
E. coli BL21 (DE3) for recombinant protein expression	Agilent	#200131
E. coli Stellar strain for In-fusion cloning	Clontech	#636763
**Chemicals, Peptides, and Recombinant Proteins**		
HisSUMO-hZFY and its truncated variants	This paper	
HisSUMO-mZFY1	This paper	
HisHALO-CTCF(F1-F9), (F1-F11), and R567W	This paper	
**Critical Commercial Assays**		
High-throughput sequencing	Illumina Miseq or Nextseq 500	
Fluorescence anisotropy, including kinetic assay	Tecan Safire 2	www.tecan.com
**Sequencing Data**		
Raw and analyzed Affinity-seq data	This paper	NCBI GEO GSE111772
Raw and analyzed Spec-seq data for ZFY	This paper	NCBI GEO GSE109098
Raw and analyzed Methyl-Spec-seq data for CTCF	This paper	NCBI GEO GSE188164
ChIP-exo data for ZIM3 and ZNF343	Trono Lab	NCBI GEO GSE78099
**Oligonucleotides**		
Randomized DNA libraries for Spec-seq experiment	This paper	ZFY Rand1-12, CTCF-R1, R2, R3, etc
Randomized DNA libraries for HT-SELEX experiment	This paper	ZFY-SELEX R1,R2
FAM-labeled ZFY-reference dsDNA probes	This paper	ZFY-reference-FAM
Unlabeled ZFY-reference competitor dsDNA	This paper	ZFY-competitor
**Recombinant DNA**		
Plasmids: HisSUMO-hZFY and its truncated variants	This paper	Stormo lab
Plasmids: Halo-tagged hZFY and mZFY1 for Affinity-seq	This paper	Petkov lab
Plasmid: HisSUMO-CTCF(F1-F9)	Addgene	Addgene #102859
Plasmid: HisHALO-CTCF(F1-F9), (F1-F11), and R567W	This paper	Fordyce lab
**Software and Analysis workflows**		
MACS	Xiaole Shirley Liu Lab	http://liulab.dfci.harvard.edu/MACS/
MEME	MEME suite	http://meme-suite.org/
Zinc finger motif prediction model	Singh Lab	zf.princeton.edu/
Zinc finger motif database	Hughes Lab	http://cisbp.ccbr.utoronto.ca
TF motif database	JASPAR	jaspar.genereg.net
TFCookbook for specificity modelling	Zheng Zuo	https://github.com/zeropin/TFCookbook
TEcookbook for repeats annotation	Zheng Zuo	https://github.com/zeropin/TECookbook
Analysis of ZFY Spec-seq data	Zheng Zuo	https://github.com/zeropin/ZFPCookbook/ZFY
Analysis of CTCF Methyl-Spec-seq data	Zheng Zuo	https://github.com/zeropin/ZFPCookbook/CTCF
ModeMap analysis of ZIM3 and ZNF343data	Zheng Zuo	https://github.com/zeropin/ZFPCookbook/ZIM3 (or ZNF343)
RCADE analysis of ChIP-exo data	Hughes lab	http://kznfmotifs.ccbr.utoronto.ca/report.php?name=ZNF343

Table S3 Key Resources Table
